# Cooperative mechanisms of oxygen vacancy stabilization and migration in the isolated tetrahedral anion Scheelite structure

**DOI:** 10.1038/s41467-018-06911-w

**Published:** 2018-10-26

**Authors:** Xiaoyan Yang, Alberto J. Fernández-Carrión, Jiehua Wang, Florence Porcher, Franck Fayon, Mathieu Allix, Xiaojun Kuang

**Affiliations:** 1 MOE Key Laboratory of New Processing Technology for Nonferrous Metals and Materials, Guangxi University Key Laboratory of Nonferrous Metal Oxide Electronic Functional Materials and Devices, College of Materials Science and Engineering, Guilin University of Technology, Guilin, Guangxi 541004 People’s Republic of China; 2 CNRS, CEMHTI UPR3079, Univ. Orléans, 45071 Orléans, France; 3 Laboratoire Léon Brillouin, CEA Saclay, 91191 Gif Sur Yvette, France

## Abstract

Tetrahedral units can transport oxide anions via interstitial or vacancy defects owing to their great deformation and rotation flexibility. Compared with interstitial defects, vacancy-mediated oxide-ion conduction in tetrahedra-based structures is more difficult and occurs rarely. The isolated tetrahedral anion Scheelite structure has showed the advantage of conducting oxygen interstitials but oxygen vacancies can hardly be introduced into Scheelite to promote the oxide ion migration. Here we demonstrate that oxygen vacancies can be stabilized in the BiVO_4_ Scheelite structure through Sr^2+^ for Bi^3+^ substitution, leading to corner-sharing V_2_O_7_ tetrahedral dimers, and migrate via a cooperative mechanism involving V_2_O_7_-dimer breaking and reforming assisted by synergic rotation and deformation of neighboring VO_4_ tetrahedra. This finding reveals the ability of Scheelite structure to transport oxide ion through vacancies or interstitials, emphasizing the possibility to develop oxide-ion conductors with parallel vacancy and interstitial doping strategies within the same tetrahedra-based structure type.

## Abstract (web summary)

Fast oxide ion conductors are the key materials for some technological devices. Here the authors report the creation and stabilization of oxygen vacancies in BiVO_4_ Scheelite with isolated tetrahedral anion structures for improved ionic conducting performance and understanding of the conduction mechanism.

## Introduction

Research for solid oxide fuel cells with lower operating temperature has stimulated the discovery of new oxide ion conductors and the understanding of the mechanisms driving the stabilization of oxide anionic defects and oxide ion mobility^1–6^. Recently, structure types containing tetrahedral moieties have received growing attention as new oxide ion conductor candidates, owing to the deformation and rotation flexibility of tetrahedral units which facilitates the stabilization and transportation of oxygen interstitial or vacancy defects^1,3,7^. Oxygen interstitials often appear as charge carriers inducing oxide ion conduction in tetrahedra-based structures^1,7–9^. Variable coordination geometry of the cations in the tetrahedral centers is important for stabilizing interstitial defects, allowing incorporation of extra oxygen atoms into the bonding environments of tetrahedral cations^1,6–9^. In low-dimensional structures, the ease of deformation and rotation of tetrahedral units allows for the accommodation and transportation of the interstitial defects. For example, La_10−*x*_(*M*O_4_)_6_O_3−1.5*x*_ (*M* = Si, Ge)-based apatites^8,10^ and La_2_Mo_2_O_9_-based (LaMOX)^2,6^ oxide ion conductors adopt isolated tetrahedral anion structures, and *Ln*_1+*x*_*A*_1−*x*_Ga_3_O_7+0.5*x*_ (*Ln* = La, Gd, Eu, Tb, *A* = Ca, Sr, Ba) gallate melilites exhibit a two-dimensional (2D) interstitial oxide ion conductivity in the layered tetrahedral network^1,11–15^.

Compared with oxygen interstitials, vacancy-mediated oxide ion conduction occurs rarely in tetrahedra-based structures. The identification of such tetrahedral structures with the ability to accommodate oxygen vacancies to facilitate oxide-on migration is important to the development of new oxide ion conductors. An example of oxygen vacancy accommodation in tetrahedra-based structures is the formation of bridging oxygen atoms between neighboring tetrahedra, which maintains tetrahedral coordination instead of producing unstable three-fold coordination geometry^3^. In such a case, the tetrahedra-based structures must possess sufficient rotation and deformation flexibility to enable a corner-sharing formation process for accommodating oxygen vacancy defects, as well as breaking of the corner-sharing structure for the oxygen vacancy migration. This mechanism was demonstrated in the case of the La_1−*x*_Ba_1+*x*_GaO_4−0.5*x*_ isolated tetrahedral anion structure^3^, one of the few oxygen vacancy conducting materials based on purely tetrahedral moieties. In La_1−*x*_Ba_1+*x*_GaO_4−0.5*x*_, the presence of oxygen vacancies leads to the formation of corner-sharing Ga_2_O_7_ units and oxygen vacancies migrate according to a cooperative mechanism involving continuous breaking and reforming of Ga_2_O_7_ dimers enabled by the rotation and deformation of Ga_2_O_7_ and GaO_4_ units^3^.

**Fig. 1:**
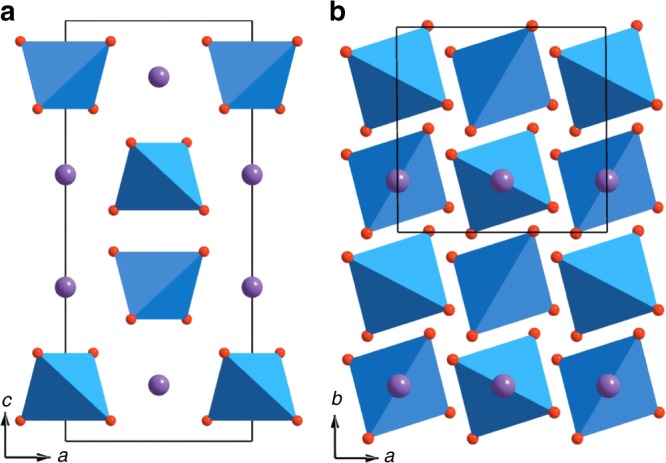
The BiVO_4_ Scheelite structure. **a** [010] and **b** [001] projections. The violet and red spheres denote Bi and O atoms, respectively. The V atoms (not shown) are at the center of the tetrahedra

Apart from tetrahedra-based structures, the advantage of tetrahedral units on transporting oxide anions has also been demonstrated in the traditional fluorite-based superstructures, for example, Bi_1−*x*_V_*x*_O_1.5+*x*_ system containing isolated VO_4_ tetrahedra diluted in the fluorite lattice, where V^5+^ cations have variable coordination numbers (from 4 to 6)^16,17^. The *x* = 0.087–0.095 composition range demonstrates high oxide ion conductivity (up to 0.04 S cm^−1^ at 500 °C) and adopts a pseudo-cubic 3 × 3 × 3 fluorite superstructure with VO_4_ tetrahedra apart from each other by ~6.7 Å without interaction between them^16^. In this superstructure, ab initio molecular dynamic (MD) simulations demonstrated that the isolated VO_4_ tetrahedra participate to transport oxide anions, which migrate by hopping among the vacancies in Bi-O fluorite slabs and are transported by the diluted VO_4_ tetrahedra through a process of continuous formation, rotation, and breaking up of highly coordinated VO_4+*n*_ polyhedra^16^. Increase of the V^5+^ content in Bi_1−*x*_V_*x*_O_1.5+*x*_ leads to closer VO_4_ tetrahedra, which constrains their rotation and thus reduces the oxide ion mobility, as revealed in Bi_46_V_8_O_89_ (*x* = 0.148)^17^. Further increase of the V content to *x* = 0.5 (i.e., to BiVO_4_) results in a Scheelite structure (Fig. 1), which is a variant of the fluorite structure but composed of isolated VO_4_ tetrahedra with all the oxygen anions bonded with V^5+^ cations. BiVO_4_ adopts a distorted Scheelite structure with monoclinic symmetry at ambient temperature and transforms to tetragonal Scheelite phase at 250 °C^18^. Even though oxide ion conductivity was reported in the BiVO_4_ Scheelite^19,20^, no further interest was devoted to the oxide ion conduction mechanism in BiVO_4_-based materials, which instead have received considerable interest for their photocatalytic properties^21,22^.

Scheelite-type materials have demonstrated interstitial oxide ion conduction, for example, donor-doped La_*x*_Pb_1−*x*_WO_4+0.5*x*_^23^ and mixed-valent CeNbO_4+*x*_^7,24^ materials, in which the oxygen interstitials are incorporated into the tetrahedral units resulting in a linked polyhedral network. Compared with the interstitial oxide ion conduction, very little attention has been paid on the oxygen vacancy conduction mechanism in Scheelites and the ability of the structure to accommodate oxygen vacancies allowing the oxide ion migration remains so far unclear. Unraveling the local structure surrounding the oxygen vacancies and elucidating the oxygen vacancy migration mechanism in the acceptor-doped Scheelites are key for the development of new oxide ion conductors with improved properties from the common Scheelite family and other related systems.

In this study, we demonstrate the creation, stabilization, and migration of oxygen vacancies in the BiVO_4_ Scheelite structure through substitution of Sr^2+^ for Bi^3+^. Multiple complementary techniques, including synchrotron powder diffraction and neutron powder diffraction (SPD and NPD), solid-state ^51^V nuclear magnetic resonance (NMR) spectroscopy, density functional theory (DFT) calculations and interatomic-potential-based MD simulations, evidence that the oxygen vacancies in Bi_1−*x*_Sr_*x*_VO_4−0.5*x*_ are accommodated via formation of corner-sharing tetrahedral V_2_O_7_ dimers and the oxygen vacancy migration in the Scheelite structure involves V_2_O_7_-dimer breaking and reforming process assisted by cooperative rotations and deformations of isolated neighboring tetrahedra.

## Results

### Bi_1−*x*_Sr_*x*_VO_4−0.5*x*_ solid solution

**Fig. 2:**
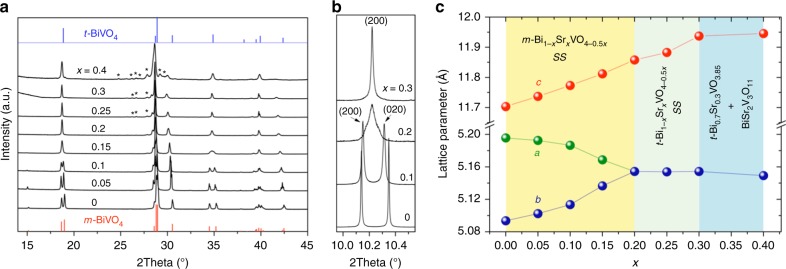
The solid solubility limit of Bi_1−*x*_Sr_*x*_VO_4−0.5*x*_. **a** Ambient temperature laboratory XRD data of Bi_1−*x*_Sr_*x*_VO_4−0.5*x*_ samples obtained by ADL synthesis. The red (bottom) and blue (top) vertical marks indicate the peak positions and relative intensities of monoclinic (no. 01-044-0081) and tetragonal (no. 01-075-2481) BiVO_4_ from the ICDD database, respectively. Asterisk marks indicate BiSr_2_V_3_O_11_ secondary phase. **b** Merging of the monoclinic (200) and (020) reflections into single (200) reflection in the tetragonal phase from high-resolution SPD data. **c** Refined cell parameters of Bi_1−*x*_Sr_*x*_VO_4−0.5*x*_ solid solution (SS). The error bars are lying behind the symbols

Initially, Bi_1−*x*_Sr_*x*_VO_4−0.5*x*_ samples were prepared by conventional solid-state (CSS) method. However, Bi_1−*x*_Sr_*x*_VO_4−0.5*x*_ (*x* = 0.05 − 0.1) samples synthesized by the CSS route form a mixture of monoclinic (*m*-Bi_1−*x*_Sr_*x*_VO_4−0.5*x*_) and tetragonal (*t*-Bi_1−*x*_Sr_*x*_VO_4−0.5*x*_) Scheelite polymorphs with the same composition, as revealed by X-ray diffraction (XRD) data analysis (Supplementary Note 1 and Supplementary Figs. 1–3). The limit of the solid solution, as *x* = 0.1 for the CSS synthesis, can be extended significantly to *x* = 0.3 (Fig. 2) via direct crystallization from the melt using aerodynamic levitation (ADL) coupled to laser heating (Supplementary Fig. 4). The ADL method provides great homogenization during the melting step and allows the stabilization of the monoclinic polymorph when the sample crystallizes during rapid cooling. Energy X-ray dispersive spectroscopy (EDS) elementary analyses performed on scanning electron microscopy (SEM) indicate highly compositional homogeneity in the Scheelite phase (Supplementary Fig. 5 and Supplementary Table 1) and neither SEM/transmission electron microscopy (TEM) nor XRD detect any remaining glass in the samples synthesized by the ADL method. The sole monoclinic polymorph is maintained up to *x* = 0.15 and the *x* = 0.2 sample forms a tetragonal phase (Fig. 2 a–b). These results are in good agreement with the compositional dependency of the cell parameters (Fig. 2c and Supplementary Fig. 6). The unit cell volume expands with the Sr^2+^ substitution for Bi^3+^, which is consistent with the larger Sr^2+^ ionic radius (the ionic radii of 8-coordinated Sr^2+^ and Bi^3+^ are 1.26 Å and 1.17 Å, respectively)^25^. The contraction of the *a*-axis and expansion of the *b*-axis are observed for *m*-Bi_1-*x*_Sr_*x*_VO_4-0.5*x*_, which result in equal *a* an *b* axes when *x* ≥ 0.2. Therefore, the Sr^2+^ substitution for Bi^3+^ appears to stabilize the tetragonal Scheelite phase. This observation is supported by variable-temperature XRD (VT-XRD) experiments performed on Bi_0.9_Sr_0.1_VO_3.95_ and the pristine BiVO_4_ samples, showing that upon heating the monoclinic-to-tetragonal phase transition temperature decreases from ~250 °C for the parent BiVO_4_ to ~150 °C for the Sr-substituted composition *x* = 0.1 (Supplementary Fig. 7). Compositions 0.25 ≤ *x* ≤ 0.4 also exhibit several extra reflections corresponding to the BiSr_2_V_3_O_11_ secondary phase^26^ (its content quantified through two-phase Rietveld refinement is ~12–15 wt% for *x* = 0.25–0.3, and then dramatically increases to ~36 wt% for *x* = 0.4, see Fig. 2a) and are related to the complex sample synthesis. For 0.2 ≤ *x* ≤ 0.3, the *a* and *b* unit cell parameters of *t*-Bi_1−*x*_Sr_*x*_VO_4−0.5*x*_ remain constant, while the *c* lattice parameter linearly increases up to 30% Sr substitution (Fig. 2c). Beyond *x* = 0.3, the lattice parameters remain unchanged, indicating the limit of the solid solution at *x* = 0.3 within tetragonal phase using the ADL approach. Owing to the ability of the ADL route to enable the synthesis of homogenous single-phase Bi_1−*x*_Sr_*x*_VO_4−0.5*x*_ samples, the following structural and property characterizations will focus on the samples obtained by ADL method (unless peculiar specification).

### Rietveld analysis

Rietveld refinements of SPD and NPD data of Bi_0.9_Sr_0.1_VO_3.95_ (Supplementary Fig. 8) were performed in order to elucidate how the acceptor Sr^2+^ substitution for Bi^3+^ and the resultant oxygen vacancies affect the BiVO_4_ Scheelite structure. The monoclinic BiVO_4_ Scheelite model^27^ described in the *I*2/*b* space group, which contains one Bi/Sr, one V and two O crystallographic sites, was used in the refinement. First, the SPD data were refined and the resulting structural model (Supplementary Table 2) obtained from the SPD data refinement was transferred to NPD data refinement, in which all positional parameters for V atoms were fixed at those obtained from the SPD data refinement given that neutrons are hardly scattered by vanadium nuclei^28^. The refinement led to a Bi_0.893(2)_Sr_0.107(2)_VO_3.918(6)_ composition, confirming the Bi_0.9_Sr_0.1_ composition and the presence of oxygen vacancies. The magnitudes of refined anisotropic atomic displacement parameters (ADPs) for the Bi/Sr and oxygen sites (Supplementary Table 3) and their thermal-ellipsoid shapes (Supplementary Fig. 9) indicate that oxygen vacancies induce positional disorder.

The difference Fourier map showed no residual scattering density that could be attributed to the presences of new crystallographic sites generated from the relaxation of atoms surrounding the vacancy defects. Therefore, the structural variation induced by the oxygen vacancy in the Sr-substituted BiVO_4_ cannot be further characterized here by the SPD or NPD techniques, which provide solely a long-range averaged model. The structural defects probably occur locally with a diversity of possible configurations, which, alternatively, solid-state NMR spectroscopy is a powerful tool to probe on the atomic scale.

### ^51^V NMR spectra and DFT calculations

To obtain insight about local structural modifications occurring upon Sr^2+^ substitution for Bi^3+^, ^51^V solid-state magic-angle spinning (MAS) NMR spectroscopy was employed as a sensitive probe of local chemical environment variation, owing to the large ^51^V chemical shift range and moderate quadrupolar interaction^29–31^. Indeed, every VO_4_ unit is surrounded by eight Bi atoms in the parent Scheelite structure and accommodation for oxygen vacancies upon Sr^2+^ for Bi^3+^ substitution should lead to significant modifications of the ^51^V chemical shift and quadrupolar coupling parameters.

**Fig. 3:**
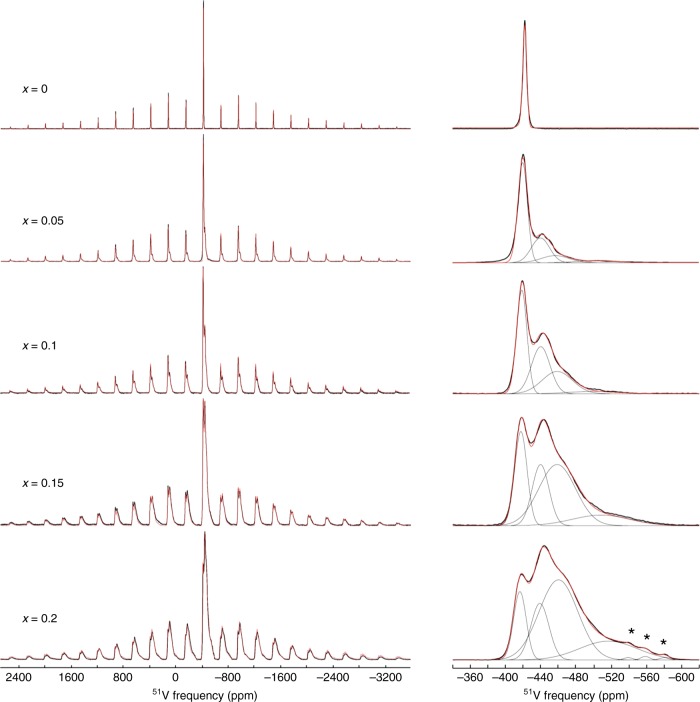
^51^V MAS NMR spectra of Bi_1−*x*_Sr_*x*_VO_4−0.5*x*_. (Left) Quantitative ^51^V MAS spectra recorded at 20.0 T with a spinning frequency of 60 kHz (black) and their corresponding best fits (red). (Right) expansion of the central-transition region. The four distinct contributions are shown as thin black lines and asterisks mark impurities (probably BiSr_2_V_3_O_11_)

Figure 3 shows the ^51^V MAS NMR spectra of Bi_1−*x*_Sr_*x*_VO_4−0.5*x*_ (*x* = 0–0.2) samples, recorded at very high magnetic field (20.0 T) and very high spinning frequency (60 kHz). The ^51^V (7/2-spin nucleus) MAS spectrum of parent BiVO_4_ exhibits a single intense central-transition (CT) resonance and spinning sideband manifolds from the corresponding six satellite transitions affected by the first-order quadrupolar interaction, thereby demonstrating the presence of a single V environment, as expected. The ^51^V isotropic chemical shift (*δ*_ISO_), quadrupolar coupling, and chemical shift anisotropy (CSA) parameters determined from the least-squares fitting of the high-field spectrum are supported by measurements performed at lower magnetic field and spinning rate (Supplementary Figs. 10–11), and are in good agreement with previous studies^30^. The ^51^V MAS spectra of Sr-substituted samples show that the Sr^2+^ substitution for Bi^3+^ in the Scheelite structure leads to the appearance of several broad ^51^V resonances, the intensities of which increase with the Sr content. The peak characteristic of BiVO_4_-like local environment (at −421 ppm) broadens and slightly shifts toward higher frequencies for increased Sr/Bi ratios. For all observed CT peaks, the linewidths (expressed in Hz) increase proportionally with the applied field (Supplementary Figs. 10–11), and strongly spread along the isotropic chemical shift distribution axis of 2D ^51^V MQMAS spectra (Supplementary Fig. 12). This demonstrates that the line broadenings are mainly due to distributions of the ^51^V isotropic chemical shift, reflecting strong positional disorder of the local chemical structures associated with each peak. Quantitative ^51^V MAS spectra were simulated considering four CT peaks of Gaussian shapes and the associated spinning sidebands manifolds from all satellite transitions. The ^51^V average isotropic chemical shift and quadrupolar coupling parameters of each contribution were determined from the least-squares fits of the spectra recorded at two magnetic fields. Due to the overlap between broad contributions, the weak effect of CSA on the intensities of the satellite-transition spinning sidebands was neglected (note that for pure BiVO_4_, neglecting the CSA effect only leads to <0.5% decrease of the quadrupolar coupling constants |*C*_Q_|). The ^51^V *δ*_ISO_, |*C*_Q_|, biaxiality parameters (*η*_Q_), and relative intensities of the four contributions corresponding to four types of vanadium local environments in Bi_1−*x*_Sr_*x*_VO_4−0.5*x*_ samples are reported in Table 1 and Supplementary Table 4.

**Table 1:** ^51^V NMR parameters

Compound	Unit		*δ*_ISO_ (ppm)	|*C*_Q_| (MHz)	*η*_Q_	*δ*_CSA_ (ppm)	*η*_CSA_	*σ*_ISO_ (ppm)	*α*, *β*, *γ* (°)
a BiVO_4_	VO_4_ (0Sr, 8Bi)	Exp.	−420.7 (5)	4.97 (2)	0.38 (5)	89 (5)	0.3 (1)		80 (20), 0, 0
		DFT	−439	5.165	0.19	62.4	0.41	−1527	26.5, 0, 0
	Experimental	***δ***_**ISO**_ **(ppm)**	|***C***_**Q**_| **(MHz)**	***η***_**Q**_	**FWHM** **(ppm)**	***I*** (% ± 5)			
b Sr_0.1_Bi_0.9_VO_3.95_	VO_4_ (0Sr, 8Bi)	−417 (1)	4.75 (5)	0.4 (1)	14 (1)	37			
	VO_4_ (1Sr, 7Bi)	−439 (3)	4.5 (1)	0.5 (1)	25 (1)	33			
	VO_4_ (2Sr, 6Bi)	−459 (5)	4.4 (2)	0.6 (2)	40 (2)	24			
	V_2_O_7_	−500 (10)	4.6 (2)	0.6 (2)	66 (8)	6			
	DFT (2 × 2 × 1) supercell	***δ***_**ISO**_^**av**^ **(ppm)**	|***C***_**Q**_|^**av**^ **(MHz)**	***η***_**Q**_^**av**^	***σ*** (***δ***_**ISO**_) **(ppm)**	***σ*** (|***C***_**Q**_|)**(MHz)**			
c Sr_0.125_Bi_0.875_VO_3.9375_	VO_4_ (0Sr, 8Bi)	−446.8	5.40	0.26	13.6	0.47			
	VO_4_ (1Sr, 7Bi)	−463.5	5.21	0.26	17.7	0.82			
	VO_4_ (2Sr, 6Bi)	−493.4	4.60	0.26	9.2	0.99			
	V_2_O_7_ (2Sr, 5–6Bi)	−558.1	8.39	0.62	11	1.19			

a ^51^V isotropic chemical shift (*δ*_ISO_), quadrupolar coupling constant (|*C*_Q_|), biaxiality of the EFG tensor (*η*_Q_), chemical shift anisotropy (*δ*_CSA_), biaxiality of the CSA tensor (*η*_CSA_), and isotropic shielding (*σ*_ISO_) determined from the experimental MAS spectra (20.0 and 9.4 T) of BiVO_4_ and from DFT-GIPAW computations after geometry optimization of the experimental structure. (*α*, *β*, *γ*) are the Euler angles describing the relative orientation of the EFG and CSA tensors. b ^51^V *δ*_ISO_, |*C*_Q_|, *η*_Q_, full-width at half-maximum (FWHM), relative intensities and assignment of the four individual contributions in ^51^V MAS NMR spectra of Sr_0.1_Bi_0.9_VO_3.95_. c Average ^51^V isotropic chemical shift (*δ*_ISO_^av^), quadrupolar coupling parameters (|*C*_Q_|^av^), and biaxiality of the EFG tensor (*η*_Q_^av^) and their standard deviations (*σ*) for isolated VO_4_ tetrahedra with different number of Sr/Bi atoms surrounding them in their second coordination spheres and V_2_O_7_ dimers in Sr_0.125_Bi_0.875_O_3.9375_

The resonance at *δ*_ISO_ −417 ppm was assigned to BiVO_4_-like local environments in Fig. 3. To identify the environments giving rise to the broader additional lines at −439, −459, and ~−500 ppm in the ^51^V MAS NMR spectra, periodic DFT calculations of the NMR parameters were performed for different possible atomic arrangements. The Gauge Included Projector Augmented Wave (GIPAW)^32^ and Projector Augmented Wave (PAW)^33^ methods, which can predict the chemical shielding and electric field gradient (EFG) tensors in a variety of solids with a high accuracy^34,35^, were employed. To create a benchmark of possible ^51^V local environments in Bi_1−*x*_Sr_*x*_VO_4−0.5*x*_ compositions, several structural models accommodating oxygen vacancies and Sr cations were considered. These models were built from a 2 × 2 × 1 supercell of the BiVO_4_ unit cell in which two Bi atoms are replaced by two Sr atoms and an oxygen atom of a VO_4_ group is removed, resulting in the formation of a V_2_O_7_ unit in the vicinity of Sr. A total of eight inequivalent supercell containing 95 atoms matching the Sr_0.125_Bi_0.875_VO_3.9375_ composition (Supplementary Fig. 13) were investigated. The DFT optimization of all atomic positions was performed with *P1* symmetry through constraining the cell parameters to the values presented Fig. 2c. The optimized 2 × 2 × 1 cells are shown in Supplementary Fig. 13. DFT geometry optimization leads to average Sr-O and Bi-O bond lengths of ~2.55 and 2.45 Å respectively, and to O-V bridging-bond lengths of ~1.82 Å, very close to the experimental values reported for BiVO_4_ and Sr_2_VO_7_. In the optimized models, which show very similar total energies, the V_2_O_7_ units are found to adopt either an almost linear or bent conformation depending on the location of Sr atoms.

**Fig. 4:**
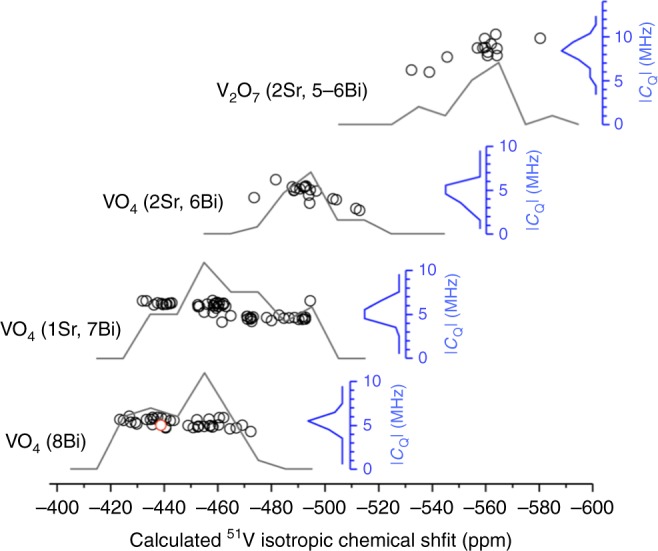
Calculated ^51^V isotropic chemical shift. Calculated ^51^V isotropic chemical shift (*δ*_ISO_) and quadrupolar coupling constants (|*C*_Q_|) for isolated VO_4_ tetrahedra with different number of Sr/Bi atom neighbors in their second coordination spheres and V_2_O_7_ dimers in Sr_0.125_Bi_0.875_VO_3.9375_. Black and blue lines correspond to histograms of calculated *δ*_ISO_ and (|*C*_Q_|), respectively

The ^51^V isotropic chemical shifts and quadrupolar coupling constants of all V sites in the 2 × 2 × 1 supercell models from the GIPAW and PAW computations are illustrated in Fig. 4. These results show that four ranges of ^51^V *δ*_ISO_ values can be identified in the 2 × 2 × 1 Sr_0.125_B_0.875_O_3.9375_ models, in good agreement with the experimental spectra. The former with an average ^51^V *δ*_ISO_ of −446.8 ppm, corresponding to BiVO_4_-like environment (i.e., isolated VO_4_ tetrahedron surrounded by eight Bi atoms), is close to the value calculated for parent BiVO_4_ (−439 ppm). Two calculated *δ*_ISO_ values centered at −463.5 and −493.4 ppm correspond to isolated VO_4_ groups with one and two Sr atoms in their second coordination spheres, respectively. Finally, the fourth calculated ^51^V *δ*_ISO_ with an average value at −558.1 ppm corresponds to the inequivalent V sites in V_2_O_7_ groups. Considering the calculated and experimental *δ*_ISO_ values for BiVO_4_ (of −439 and 419 ppm, respectively) as internal references, the computational results nicely mimic the experimental trends and the four contributions at −417, −439, −459, and −500 ppm in the experimental MAS NMR spectra can be confidently assigned to isolated VO_4_ groups with 0, 1, and 2Sr neighbors and to V_2_O_7_ units, respectively. Calculated average |*C*_Q_| values are also in good agreement with experimental values for isolated VO_4_ groups, while overestimated values are obtained for the V_2_O_7_ units, possibly due to the limited number of V_2_O_7_ local conformations considered in the structural models (1 V_2_O_7_ dimer for a 2 × 2 × 1 supercell of Sr_0.125_Bi_0.875_VO_3.9375_). Following the above line assignment, the amount of V atoms involved in V_2_O_7_ units determined from MAS spectra are 4, 6, 16, and 20% for *x* = 0.05, 0.1, 0.15, and 0.2, respectively, very close to the values expected from sample compositions (5, 10, 15, and 20%).

Clearly, both experimental and computational NMR results indicate that generation of oxygen vacancies in Bi_1−*x*_Sr_*x*_O_4−0.5*x*_ Scheelite structure occurs via formation of bridging V–O–V bonds to avoid the formation of unstable three-fold coordinated V centers.

### Ionic conductivity

**Fig. 5:**
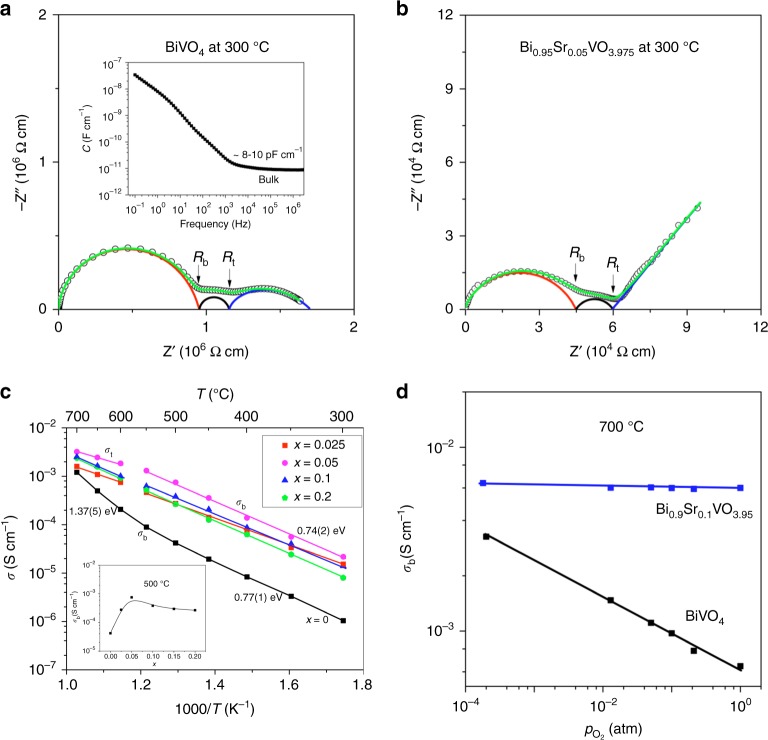
Ionic conductivity. Typical complex impedance plots at 300 °C of **a** BiVO_4_ and **b** Bi_0.95_Sr_0.05_VO_3.975_ and their fitted curves (green), as well as the deconvolution of bulk (red), grain boundary (black), and electrode (blue)-response arcs. *R*_b_ and *R*_t_ denote bulk and total resistivities, respectively. The inset in **a** shows the capacitance as a function of frequency. **c** Arrhenius plots of the conductivities for Bi_1−*x*_Sr_*x*_VO_4−0.5*x*_ (*x* = 0–0.2). The inset shows the bulk conductivity at 500 °C as a function of *x*. **d** The *p*O_2_ dependency of the conductivity of BiVO_4_ and Bi_0.9_Sr_0.1_VO_3.95_ at 700 °C

Figure 5a shows the complex impedance plot of parent BiVO_4_ at 300 °C, which comprises bulk, grain boundary, and electrode responses. The large semicircular arc in the high-frequency region displays capacitances of ~8–10 pF cm^−1^ (inset of Fig. 5a), ascribed to the bulk response^36^, which may be simply modeled by a parallel resistor (*R*_b_) and capacitor (*C*_b_). The intercept of the large semicircular arc at low frequency was extracted as the bulk resistivity *R*_b_ and the associated *C*_b_ calculated by using the equation *ω*RC = 1 (*ω* = 2π*f*_max_, where *f*_max_ is the frequency at maximum imaginary impedance $Z''_{\max }$; *ω* is also equal to the inverse of the relaxation time *τ*) is 10 pF cm^−1^. This value is close to that calculated using $M''_{\max } = \varepsilon _0/2{C}$^37^, where $M''_{\max }$ is the maximum imaginary modulus for this semicircular arc. This indicates that the imaginary impedance and modulus peaks (Supplementary Fig. 14) in the high-frequency region are associated with the same RC elements, that is, that the large semicircular arc can be ascribed to a single bulk response^37^. The grain boundary response overlaps significantly with the electrode response, giving an essentially flattened/depressed arc in the low-frequency region. The deconvolution of the impedance plot for BiVO_4_ at 300 °C, performed through the equivalent circuit fitting with bulk, grain boundary, and electrode components (see Supplementary Note 2 for details), is shown in Fig. 5a. The large capacitance of ~10^−8^–10^−7^ F cm^−1^ in the 10–0.1 Hz low-frequency region is indicative of ionic conduction in parent BiVO_4_^36^. With the increase of temperature, the bulk response arc gradually disappears, which results in the mixed grain boundary and electrode-response arcs observed at 700 °C (Supplementary Fig. 15a).

The impedance data for the strontium-substituted compositions contain significant Warburg-type electrode response in addition to the bulk and grain boundary responses (Fig. 5b), suggesting that ionic conduction is enhanced by the Sr substitution. Figure 5b shows the complex impedance plot and its deconvolution for a Bi_0.95_Sr_0.05_VO_3.975_ pellet at 300 °C. With the increase of temperature, both bulk and grain boundary responses gradually disappear and the electrode response collapsed down to semicircular arc at high temperature in air (Supplementary Fig. 15b and Supplementary Fig. 16a). Under low partial oxygen pressure (*p*O_2_) N_2_ environment, the electrode-response arc reverted back to a Warburg-type spike characteristic of oxide ion conduction (Supplementary Fig. 16a), in contrast with the parent BiVO_4_ pellet for which the electrode-response arc remained as a semicircular arc shape (Supplementary Fig. 16b). Such a behavior, for the Sr-substituted compositions of the electrode-response arc versus *p*O_2_, is consistent with the *p*O_2_-controlled kinetics of the oxygen ion diffusion and charge transfer reaction of O_2_ + 2e ↔ 2O^2−^ along the sample–electrode–gas interface, similar to common observations in oxide ion conductors^38–40^. Thus, the oxide ion conductivity is enhanced by the Sr substitution for Bi in BiVO_4_.

The Arrhenius plots of bulk conductivities (Fig. 5c) for Bi_1−*x*_Sr_*x*_VO_4−0.5*x*_ show a conductivity increase by at least one order of magnitude in the Sr-substituted compositions compared with the parent material below 600 °C. The conductivity of the parent BiVO_4_ varies within 10^−6^–10^−4^ S cm^−1^ between 300 and 600 °C. The *x* = 0.05 composition displays the highest conductivity (10^−5^–10^−3^ S cm^−1^) among the substituted compositions in the same temperature region and an activation energy (*E*_a_) of 0.74(2) eV, close to 0.77(1) eV for BiVO_4_. The parent BiVO_4_ displays significant increase of *E*_a_ to 1.37(5) eV above 600 °C, which led to a conductivity at 700 °C for BiVO_4_ almost matching those of the Sr-substituted compositions (Fig. 5c). In order to confirm the nature of the charge carriers, oxygen transport numbers ($t_{\mathrm{O}^{2 - }}$) in Bi_1−*x*_Sr_*x*_VO_4−0.5*x*_ (*x* = 0, 1) were determined via electromotive force (EMF) measurements of oxygen concentration cells within 500–700 °C. Owing to the large-size samples required for the EMF measurements compared to the size limitation of the samples synthesized by the ADL method, the *x* = 0 and 0.1 pellets from the CSS route were used in the oxygen concentration cells, although the *x* = 0.1 sample is composed of a mixture of the monoclinic and tetragonal polymorphs. As indicated by the VT-XRD data (Supplementary Fig. 17), this two-polymorph system eventually transformed to single tetragonal polymorph above 150 °C, similarly to the single-monoclinic-phase sample *x* = 0.1 synthesized from the ADL method (Supplementary Fig. 7a, c). Therefore, the oxygen transport numbers measured at high temperatures on the *x* = 0.1 pellet from the CSS route are indeed referring to single tetragonal phase, similarly to those for the pristine BiVO_4_ material synthesized from the CSS route, as well as the conductivity properties of the single-phase Bi_1−*x*_Sr_*x*_VO_4−0.5*x*_ samples obtained from the ADL method that were recorded above the phase transition temperatures.

The parent BiVO_4_ has a $t_{\mathrm{O}^{2-}}$ value ~0.27 at 500 °C, which decreases down by ~50% to 0.12 at 700 °C. This confirms mixed electronic–ionic conduction in BiVO_4_ and suggests that the electronic conduction becomes more dominative over the oxide ionic conduction at elevated temperature, which could explain the increase of *E*_a_ for the parent BiVO_4_ at >600 °C (Fig. 5c). The *p*O_2_ dependency of the conductivity at 700 °C (Fig. 5d) suggests n-type electronic conduction in the parent BiVO_4_. The existence of a low level of oxide ion conduction in the parent BiVO_4_ could be ascribed to the possible uncontrollable Bi deficiency owing to the volatilization of Bi_2_O_3_ during the material preparation, which could result in the presence of oxygen vacancies. Bi nonstoichiometry effect on the oxide ion conduction has been demonstrated in Na_0.5_Bi_0.5_TiO_3_-based oxide ion conductors^38,39,41^.

Bi_0.9_Sr_0.1_VO_3.95_ has $t_{\mathrm{O}^{2-}}$ values ~0.71–0.88 within 500–700 °C, which together with the enhanced conductivities (Fig. 5c) and the electrode-response–arc behavior of *p*O_2_ (Supplementary Fig. 16a) confirm that the oxide ionic conduction in BiVO_4_ is apparently enhanced upon the Sr^2+^ substitution for Bi^3+^. No significant changes of $t_{\mathrm{O}^{2-}}$ were observed as a function of the temperature and the conductivity of Bi_0.9_Sr_0.1_VO_3.95_ at 700 °C is essentially *p*O_2_-independent (Fig. 5d), indicating that in the Sr-substituted compositions the oxide ionic conduction predominates over the electronic conduction for the measured temperature region in high pO_2_ atmospheres.

### Oxygen vacancy migration

**Fig. 6:**
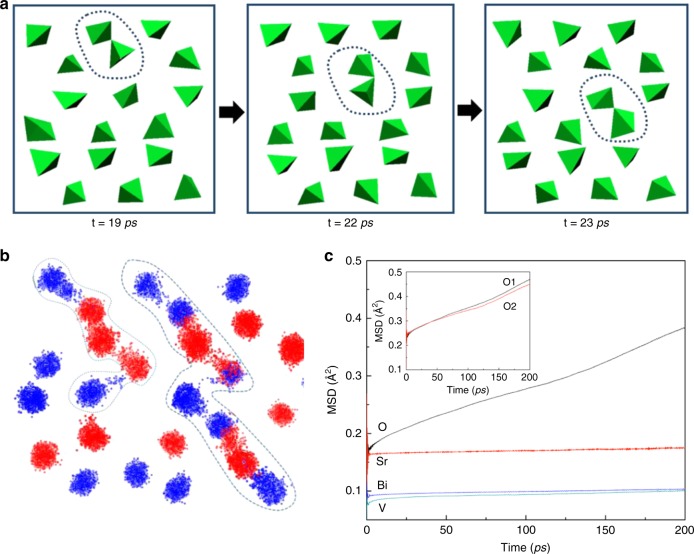
Oxygen vacancy migration in Sr_0.0625_Bi_0.9375_VO_3.96875_. **a** Snapshots from MD simulation at 1400 °C showing long-range oxygen vacancy migration involving V_2_O_7_-dimer breaking and reforming. **b** Scatter plot of oxide ion positions roughly corresponding to the dimension in **a** from MD simulation. The V_2_O_7_-dimer breaking and reforming results in long-range diffusion paths (marked by dashed lines). Blue and red dots denote oxide ions originally at O1 and O2 sites, respectively. The Bi, Sr, V, and some O ions are omitted for clarity. **c** Calculated MSD values of Sr, Bi, V, and O atoms as function of time from the MD simulation at 1400 °C. The inset shows the MSD values for O atoms originally at sites O1 and O2, which are close to each other indicating that both oxygen sites participate equally in the oxide ion migration

In order to elucidate how the oxygen vacancies migrate in the Scheelite structure, atomistic static lattice and MD simulations based on the interatomic potential method were performed, as previously successfully applied in other structure types based on tetrahedral units, for example, apatite-based^9^, melilite-based^12^, and LaBaGaO_4_-based^3^ oxide ion conductors. The potential parameters listed in Supplementary Table 5 well reproduce the Scheelite BiVO_4_ structure. The differences between calculated and experimental cell parameters and bond lengths (Supplementary Table 6) are < ±0.06 and ±0.2 Å, respectively. It is interesting to note that the calculated BiVO_4_ structure appears as a tetragonal structure (Supplementary Table 6). Given that the conductivity data described above correspond to the high-temperature tetragonal phase of Bi_1−*x*_Sr_*x*_VO_4−0.5*x*_, the calculated BiVO_4_ structure led to an excellent structural model for the MD simulation of oxygen vacancy migration in the Sr-substituted BiVO_4_, although the weak distortion of VO_4_ tetrahedra in the room temperature monoclinic Scheelite structure is not reproduced. The calculated solution energy of Sr^2+^ on Bi^3+^ site via the defect reaction $2{\mathrm{SrO}} + 2{\mathrm{Bi}}_{\mathrm{Bi}}^ \times + {\mathrm{O}}_{\mathrm{O}}^ \times \to 2{\mathrm{Sr}}_{\mathrm{Bi}}^\prime + {\mathrm{V}}_{\mathrm{O}}^{ \cdot \cdot } + {\mathrm{Bi}}_2{\mathrm{O}}_3$ is ~1.67 eV, which is consistent with the solubility of Sr^2+^ in Bi^3+^ site in the BiVO_4_ Scheelite. MD simulations indicate that in the Sr-substituted BiVO_4_ Scheelite materials, the long-range migration of oxygen vacancies (Fig. 6a and Supplementary video file) takes place via the continuous breaking and reforming of V_2_O_7_ dimer. Such an oxygen vacancy migration is allowed by a cooperative mechanism involving the synergic rotation and deformation of neighboring VO_4_ tetrahedra that transfer oxide anions between V_2_O_7_ and VO_4_ units, thus allowing the V_2_O_7_-dimer breaking and reforming process, akin to La_1−*x*_Ba_1+*x*_GaO_4−0.5*x*_ case^3^. The scatter plot of oxygen ions (Fig. 6b) shows long-range diffusion paths in Sr-substituted BiVO_4_ arising from V_2_O_7_-dimer breaking and reforming. The streaming and overlapping of colors of oxygen ions in the scatter plot and the mean square displacement (MSD) plots (Fig. 6c) suggest that both distinctly crystallographic oxygen sites O1 and O2 are involved in the oxide ion migration. This observation is consistent with the cooperative rotation and deformation of VO_4_ tetrahedra required for exchanging the oxide anions, and in agreement with the results from Rietveld refinements, which indicate the presence of oxygen vacancies in both oxygen sites. In contrast, heavier Bi^3+^, Sr^2+^, and V^5+^ cations vibrate around their lattice positions without long-range migration (Fig. 6c). The MSD values of oxygen atoms were used to calculate the oxygen diffusion coefficient, which is estimated as ~2 × 10^−7^–7 × 10^−8^ cm^2^ s^−1^ within the 1200–1600 °C temperature range, although no experimental values are available for comparison. The activation energy of ~0.57 eV derived from the Arrhenius plot of the calculated oxygen diffusion coefficients (Supplementary Fig. 18) is lower than experimental values from the conductivity measurements (~0.74 eV).

The ability to accommodate and transport the oxygen vacancies demonstrated in Bi_1−*x*_Sr_*x*_VO_4−0.5*x*_ make the BiVO_4_ Scheelite structure unique when compared with other reported Scheelite compositions which can hardly accommodate mobile oxygen vacancies. The fluorite-like Bi-O sublattice, which is known as a highly polarizable bonding network^16,38,41^, could offer flexible environments to adapt for rotation and deformation of VO_4_ tetrahedra, which is important for the stabilization and migration of oxygen vacancies in a tetrahedra-based structure. Therefore, the Bi-O lattice provides pathways with low diffusion barriers, which are highly beneficial for the migration of oxygen ions. The creation of oxygen vacancies through Sr substitution for Bi could be offset by decreasing the concentration of Bi-O bonds owing to the decrease of the metal–oxygen polarizable bonds, which could hinder further improvement on the ionic conductivity when more Bi^3+^ is replaced with Sr^2+^ in BiVO_4_ beyond the *x* = 0.05 composition. Alternatively, the lower conductivity when *x* > 0.05 compositions could be linked to the structural distortion or to the interaction between defects.

The fact that an optimal ionic conductivity can be observed for an intermediate substitution content, or oxide ion defect concentration, within a solid solution has been widely observed in oxide ion conductors^15,42–44^. Under high substitution concentration, interactions between the defects can act as traps for the migrating oxide ions, thereby reducing the oxide ion mobility and therefore lowering the conductivity when the substitution concentration exceeds the optimal value^42,43^. In the case of Sr-substituted BiVO_4_, the calculated association energies for ${\mathrm{Sr}}_{\mathrm{Bi}}^\prime + {\mathrm{V}}_{\mathrm{O}}^{ \cdot \cdot }$ and $2{\mathrm{Sr}}_{\mathrm{Bi}}^\prime + {\mathrm{V}}_{\mathrm{O}}^{ \cdot \cdot }$ defect complexes are, respectively, 0.41 and 0.57 eV, confirming that when the Sr-substitution concentration increases, more trapping effect takes place on the vacancy migration from the defect interaction, although these energies imply that the trapping of oxygen vacancies is not significant in Sr-substituted BiVO_4_. The vacancy migration in Bi_1–*x*_Sr_*x*_VO_4–0.5*x*_ requires cooperation between a V_2_O_7_ dimer and a neighboring VO_4_. Therefore, the increase of oxygen vacancies will reduce the number of VO_4_ tetrahedral units around the V_2_O_7_ dimer. Ultimately, the accumulation of V_2_O_7_ defect units will block the vacancy migration. It is worth noting that increasing the Sr/Bi ratio and concentration of V_2_O_7_ in BiSr_2_V_3_O_11_ present as a secondary phase^26^ for *x* > 0.3 (Fig. 2a), which contains ordered V_2_O_7_ and VO_4_ units in a 1:1 ratio, results in a significant dampening of the ionic conductivity (Supplementary Fig. 19). This fact further emphasizes the importance of a Bi-O lattice and optimal oxygen vacancy content for oxide ion conduction in Sr-substituted BiVO_4_ Scheelite. Apart from the defect interaction and composition change, the possible existence of this insulating impurity below detection limits could also contribute to the conductivity degradation at the highly Sr-substituted compositions but such an effect is expected to be limited.

Although our work dedicates to the oxygen vacancy defect chemistry and vacancy-mediated oxide ion migration in the BiVO_4_ Scheelite, the fine details and understanding of the defect chemistry and structure in this work could also be useful for understanding other physical properties related to the acceptor–dopant character of BiVO_4_ Scheelite, for example, its well-known visible-light photocatalytic activity for the degradation of organic pollutants and water oxidation^21,22^.

## Discussion

Sr^2+^ substitution for Bi^3+^ in BiVO_4_ leads to the generation and migration of oxygen vacancies in the isolated tetrahedral anion Scheelite structure. The solid solution limit in Bi_1−*x*_Sr_*x*_VO_4−0.5*x*_ can be extended significantly from *x* = 0.1 using solid-state reaction synthesis to *x* = 0.3 via direct crystallization from the melt, using ADL coupled to laser heating system as an original elaboration method. Rietveld analysis performed on powder diffraction data reveals positional disorder and the presence of oxygen vacancies in Bi_1−*x*_Sr_*x*_VO_4−0.5*x*_. Solid-state ^51^V NMR spectroscopy coupled to DFT calculations allowed a precise assignment of ^51^V chemical shifts to the local structures, which demonstrate that the oxygen vacancies are accommodated via the formation of corner-sharing V_2_O_7_ tetrahedral dimers. The Sr^2+^ substitution reduces the monoclinic-to-tetragonal phase transition temperature of BiVO_4_ and significantly enhances the oxide ion conductivity by ~1–2 orders of magnitude. MD simulations indicates that oxygen vacancies migrate via a cooperative mechanism allowed by the synergic rotation and deformation of neighboring VO_4_ tetrahedra that assist the continuous breaking and reforming of V_2_O_7_ dimers. This oxygen vacancy conducting Bi_1−*x*_Sr_*x*_VO_4−0.5*x*_ Scheelite material reported here, along with the known interstitial oxide ion conducting features of Scheelites, demonstrate that the Scheelite structure is able to incorporate oxygen vacancy or interstitial defects to facilitate high oxide ion migration. This suggests that parallel vacancy and interstitial doping strategies to the same tetrahedra-based structure type can be employed for discovery of new oxide ion conductors in the future.

## Methods

### Synthesis

The synthesis of BiVO_4_ and Sr-substituted Bi_1−*x*_Sr_*x*_VO_4−0.5*x*_ compositions was carried out by the CSS method and ADL^45^ coupled to laser heating method, which allowed extending the solid solution range in Bi_1−*x*_Sr_*x*_VO_4−0.5*x*_ beyond the limit reached in CSS route via a two-step method: first melting (enabling strong homogenization) and then crystallizing the melt upon rapid cooling (by switching off the lasers)^46^.

For the CSS synthesis, Bi_2_O_3_ (99.9%), NH_4_VO_3_ (99%), and SrCO_3_ (99%) starting materials were weighed according to the nominal Bi_1−*x*_Sr_*x*_VO_4−0.5*x*_ (*x* = 0–0.2) compositions and mixed in ethanol in an agate mortar. The dried powders were pressed into pellets (12 mm diameter and ~2 mm thick) under a uniaxial pressure of 330 MPa and calcined at 600 °C for 5 h in air with heating and cooling rates of 5 °C min^−1^. The calcined pellets after the first step were ground and pressed once more for a final sintering step at 700 °C for 5 h.

Regarding the ADL synthesis, stoichiometric amounts of the same starting materials were weighed and homogenized in an agate mortar using ethanol. The powder mixtures were then dried and pressed into pellets. Pieces of the powder pellets were then placed onto a metallic nozzle and were levitated under an oxygen flow. Two CO_2_ lasers were used to homogeneously melt the samples from top and bottom (see Supplementary Fig. 4). The melt was then left to homogenize for 30 s. Polycrystalline bead materials (3 mm of diameter) were then obtained by crystallization from the melt by decreasing the power of the two laser beams^45,47^. The compositional homogeneity of the samples were analyzed by SEM and EDS using an FEI ESEM XL 40 apparatus.

### Powder diffraction

The identification of the crystalline phases was performed by powder XRD using a Panalytical X’Pert diffractometer equipped with Cu Kα radiation source. VT-XRD data were collected over the 10–80° 2*θ* range at every 50 °C from room temperature up to 800 °C during both heating and cooling steps, allowing 3 min for temperature equilibration before collecting the data sets for 8 min. Laboratory XRD data were analyzed using the Topas-Academic software^48^.

High-intensity and high-resolution SPD data were recorded on the 11BM diffractometer at the Advanced Photon Source, Argonne National Laboratory. SPD data were collected over the 0.5–40° 2*θ* range with a 0.001° step size at room temperature using λ = 0.4593 Å. In order to avoid important absorption effects from the sample, which could hinder fine structural analysis, Si-glass capillaries were coated with a layer of sample powder mixed with silicone grease. The Si-glass capillaries were then introduced in Kapton capillaries which were sealed by clay. On the other hand, constant-wavelength (λ = 1.225 Å) NPD data were collected at ambient temperature over the 10–120° 2*θ* range at 2*θ* intervals of 0.05° on the 3T2 diffractometer at Laboratoire Leon Brillouin (France). The large volume of Bi_0.9_Sr_0.1_VO_3.95_ powdered sample for neutron diffraction was obtained by the combination of multiple beads from ADL route that were grounded and prepared in a vanadium can. Rietveld analysis of SPD and NPD data was performed using the Jana2006 package^49^.

### Solid-state NMR

^51^V solid-state NMR experiments were performed on Bruker Avance III spectrometers operating at magnetic fields of 20.0 and 9.4 T (corresponding ^51^V Larmor frequencies of 223.6 and 105.2 MHz) using Bruker 1.3 and 2.5 mm MAS probe heads. ^51^V quantitative MAS spectra of the Bi_1-*x*_Sr_*x*_VO_4-0.5*x*_ samples were recorded at spinning frequencies of 60 kHz (20.0 T) and 30 kHz (9.4 T), using short pulse lengths corresponding to a 9° flip angle^50^ (0.25 µs with *ν*_nut_ = 167 kHz at 20.0 T and 0.50 µs with *ν*_nut_ = 50 kHz at 9.4 T) and a recycle delay of 1 s. Triple-quantum magic-angle spinning 2D spectra were recorded at 9.4 T (spinning frequency of 30 kHz) using the Z-filter sequence^51^. Triple-quantum coherence excitation and reconversion were performed with a nutation frequency of 45 kHz. Eighty rotor-synchronized *t*_1_ time increments were recorded with 672 transients each using a recycle delay of 1 s. ^51^V chemical shifts were referenced relative to neat VOCl_3_ (0 ppm) using a 0.16 M NaVO_3_ aqueous solution as secondary standard (−574.4 ppm)^52^. ^51^V chemical shifts and quadrupolar coupling parameters of each resonance were determined from fits of the whole spinning sideband manifold observed for all seven ^51^V transitions in MAS spectra^53,54^ using the DMfit program^55^.

### Ionic conductivity measurement

Alternating-current (AC) impedance spectroscopy measurements were performed with a Solartron 1260 frequency response analyzer over a frequency range of 10^−1^–10^7^ Hz within the 100–700 °C temperature range. Gold paste was coated on the opposite faces of the pellets and heat treated at 550 °C for 40 min to burn out the organic components in the paste to form gold electrodes. The impedance data analysis was carried out using the ZView software^56^. The oxygen transport number was determined by EMF measurements on oxygen concentration cells^57^ of O_2_||air and N_2_||air at 500–700 °C. The pellets were attached on the alumina tube using a glass sealant heat treated at 750 °C. The gas tightness was examined by using soapy water after cooling down to room temperature to ensure the absence of gas leakage prior to high temperature EMF measurements. The theoretical EMF values of the oxygen concentration cells were calculated using the Nernst equation. The partial oxygen pressure (*p*O_2_) in the N_2_ gas was determined as 10^−4^ atm by an YSZ sensor at 800 °C. The oxygen transport numbers were calculated from the ratio of experimental and theoretical EMF values. The AC conductivities as a function of *p*O_2_ were measured at 700 °C over a high *p*O_2_ range of 10^−4^–1 atm, monitored by the YSZ sensor that is placed close to the sample. The *p*O_2_ was controlled using O_2_–N_2_ (within 1–10^−4^ atm) gas mixtures, for which the HORIBA mass flow controllers (S48 32/HMT) were used. Use of lower-*p*O_2_ gas resulted in decomposition of Bi_1 − *x*_Sr_*x*_VO_4 − 0.5*x*_.

### DFT calculation

DFT calculations of the ^51^V chemical magnetic shielding and EFG tensors with periodic boundary conditions were performed with the CASTEP (8.0) code^58^. The PAW^33^ and GIPAW^32^ methods were employed for computing the EFG and NMR chemical shielding tensors, respectively. Electron correlation effects were described using the Perdew–Burke–Ernzerhof generalized gradient approximation^59^ and the core–valence interactions were described by ultrasoft pseudopotentials (USPPs)^60^ generated using the on-the-fly generator included in the CASTEP. Further descriptions of the pseudopotentials employed are given in Supplementary Table 7. For all computations, an energy cut-off of 600 eV was used for the plane wave basis set expansion and the Brillouin zone was sampled using a Monkhorst–Pack grid spacing of 0.04 Å^–1^. Computations for the Bi_0.875_Sr_0.125_VO_3.9375_ 2 × 2 × 1 supercell models (95 atoms) were performed after DFT optimization of atomic positions (*P1* symmetry with cell parameters fixed to Rietveld refined values with a very high accuracy) using the same USPP and computational parameters. The ^51^V isotropic chemical shifts (*δ*_ISO_) were deduced from calculated isotropic shielding values (*σ*_ISO_) using the relationship *δ*_ISO_ = −0.95(10) *σ*_ISO_ − 1889(145), which accounts for systematic errors on calculated values using this method. The relationship was obtained from the linear regression between experimental *δ*_ISO_ and calculated *σ*_ISO_ values for a series of 11 crystalline vanadate compounds of known structures (Supplementary Table 8 and Supplementary Fig. 20). For all reference compounds, DFT optimization of atomic positions (keeping symmetry constraints with cell parameters fixed to experimental values) was performed prior to GIPAW calculations. Computational parameters were the same as those described above.

### Static lattice and MD simulations

The atomistic static lattice simulation based on the interatomic-potential approach^61^ for calculation of defect formation energy in the BiVO_4_ Scheelite structure was performed using the General Utility Lattice Program (GULP)^62^. The Buckingham potential function^63^ was used to describe the interactions between ions with the shell model^63^ to describe the electronic polarizability for the structure modeling of BiVO_4_. The potential parameters used for the atomistic simulation of Sr-substituted BiVO_4_ are described in Supplementary Table 5. The Bi^3+^-O^2−^, Sr^2+^-O^2−^, and V^5+^-O^2−^ potential parameters were taken from previous studies^64–66^. Bi^3+^-Bi^3+^ and O^2−^-O^2−^ potential parameters were obtained through the relaxed fitting procedure implemented in the GULP code based on the initial parameters from Abrahams et al.^64^ via refining the *A* values only during the fitting for a better reproduction of the BiVO_4_ Scheelite structure. The extrinsic and intrinsic defect formation energies were then calculated based on appropriate combinations of vacancy, interstitial, and dopant defect energy terms. The oxygen vacancy migration mechanism in Sr-substituted BiVO_4_ was investigated by interatomic-potential-based MD simulations, which were performed with the DL_POLY code^67^. The simulation box consisted of 8 × 8 × 4 unit cells containing 6112 atoms and corresponding to a Sr_0.0625_Bi_0.9375_VO_3.96875_ composition. The Sr dopants and oxygen vacancies were distributed randomly within the simulation box. The systems were equilibrated first under a constant pressure of 1 atm at specific temperatures between 1200 °C and 1600 °C for 750,000 time steps with a time step of 0.2 fs before carrying out the main MD simulation for 300 ps with 1.5 × 10^6^ time steps in the NVT ensemble. The Visual MD package^68^ was used to perform MD data analysis and the MSDs were calculated with the nMoldyn code^69^. Oxygen diffusion coefficients are calculated from the slope of the MSD plots as a function of simulation time.

## Electronic supplementary material

Supplementary Information

Description of Additional Supplementary Files

Supplementary Movie 1

## Data Availability

All relevant data supporting the findings of this study are available from the corresponding authors upon request.
