# Supplementary material for: Cooperative mechanisms of oxygen vacancy stabilization and migration in the isolated tetrahedral anion Scheelite structure
Source: Nat Commun. 2018 Oct 26;9:4484. doi: 10.1038/s41467-018-06911-w (PMC6203716; doi:10.1038/s41467-018-06911-w)
Supplement: Supplementary file 1 — Supplementary Information [file 41467_2018_6911_MOESM1_ESM.pdf]

Supplementary information

Cooperative Mechanisms of Oxygen-Vacancy Stabilization and Migration in the Isolated Tetrahedral Anion Scheelite Structure

Yang et al.

Supplementary Figures

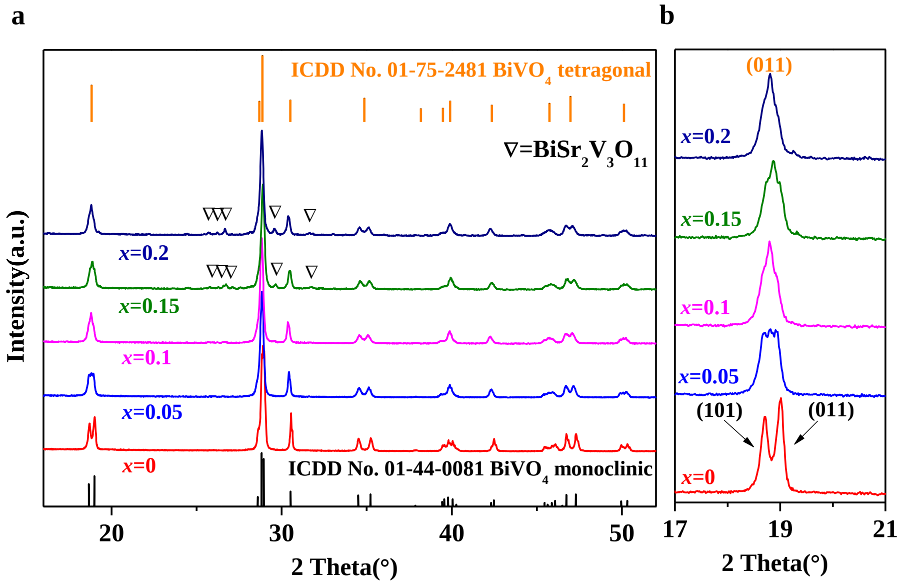

Supplementary Figure 1. $Bi_{1-x}Sr_xVO_{4-0.5x}$ Solid solution prepared by the CSS method. (a) Ambient temperature XRD data of $Bi_{1-x}Sr_xVO_{4-0.5x}$ samples synthesized from the CSS method. (b) Enlargements of the plots within the 2θ range of 17–21° showing the mergence trend of the monoclinic (101) and (011) reflections to single tetragonal (011) reflection versus the Sr-content increase (note that the Scheelite phases with compositions x = 0.05–0.2 are mixed monoclinic and tetragonal polymorphs). The symbols ∇ denote the reflections corresponding to the $BiSr_2V_3O_{11}$ secondary phase.

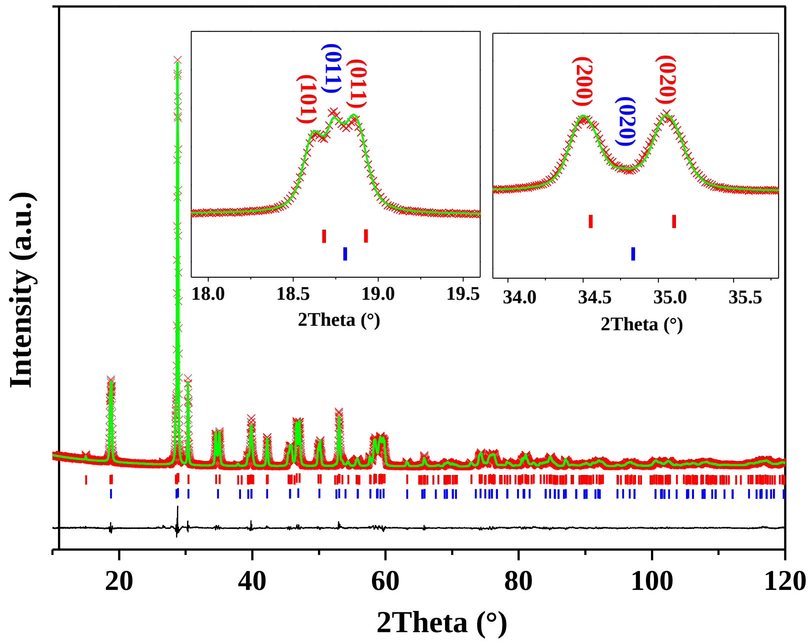

Supplementary Figure 2. Two-phase Rietveld refinement. Rietveld plot of XRD data for $Bi_{0.9}Sr_{0.1}VO_{3.95}$ composition (R_p ~ 4.42 %; R_{wp} = 5.79%; *GOF* = 2.16). The insets enlarge the fits within 18–19.5° and 34.0–3.5° 2Theta ranges. The (*hkl*) indices for the reflections of the monoclinic (red) and tetragonal (blue) scheelite phases are labeled in the insets. The red and blue vertical ticks denote Bragg reflection positions for the monoclinic and tetragonal Scheelites, respectively. The Stephens approach[1] combined with a Voigt function was employed to model the diffraction peaks that exhibit evident anisotropic peak broadening e.g., (200) and (020) reflections for the monoclinic phase and (020) reflection for the tetragonal phase in the right inset.

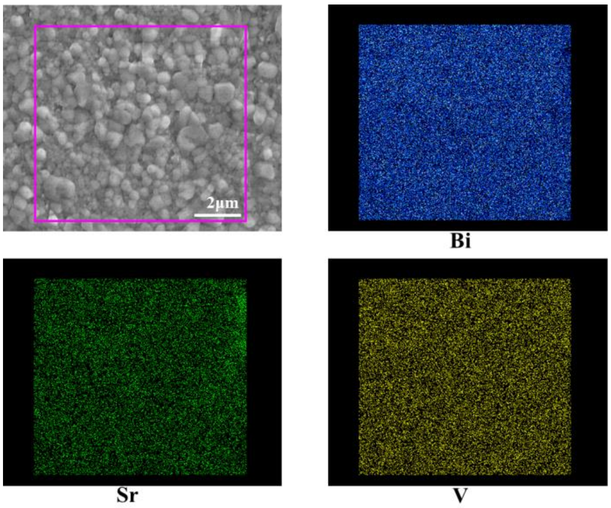

Supplementary Figure 3. Microscale chemical homogeneity. Typical SEM-EDS elemental mapping results of the mixed monoclinic and tetragonal polymorph of 10% Sr-substituted $BiVO_4$ pellet synthesized via the CSS method.

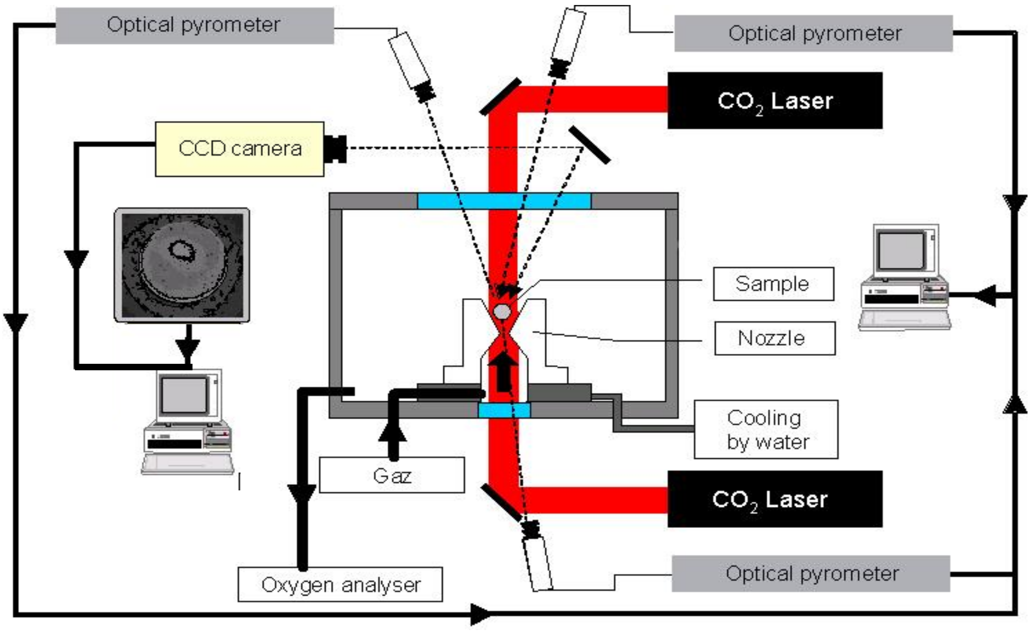

Supplementary Figure 4. Aerodynamic Levitator System. Scheme of the aerodynamic levitator (ADL) system coupled with two CO_2 laser beams.[2]

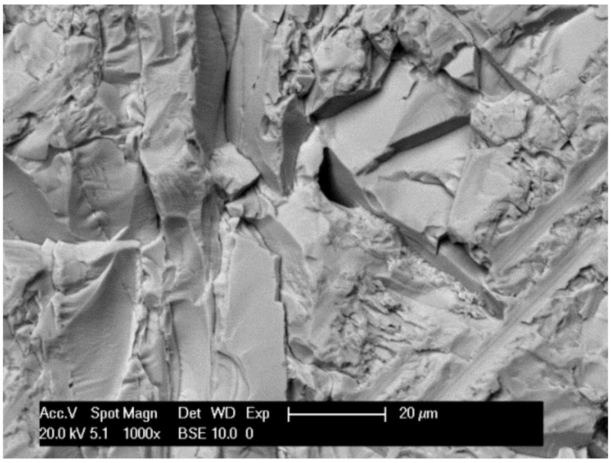

Supplementary Figure 5. SEM micrograph. Typical SEM image of the $Bi_{0.9}Sr_{0.1}VO_{3.95}$ material prepared by the ADL method.

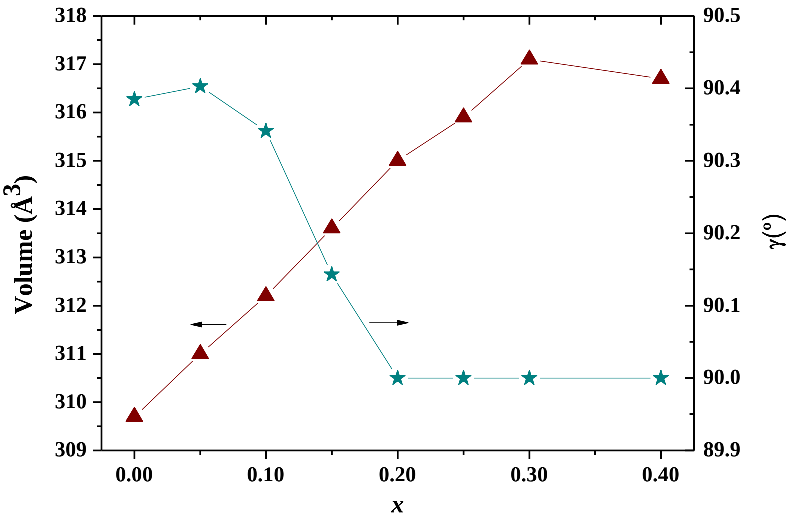

Supplementary Figure 6. Compositional dependency of the cell parameters. The refined cell parameters (Volume and γ) of $Bi_{1-x}Sr_xVO_{4-0.5x}$ samples obtained from the ADL method.

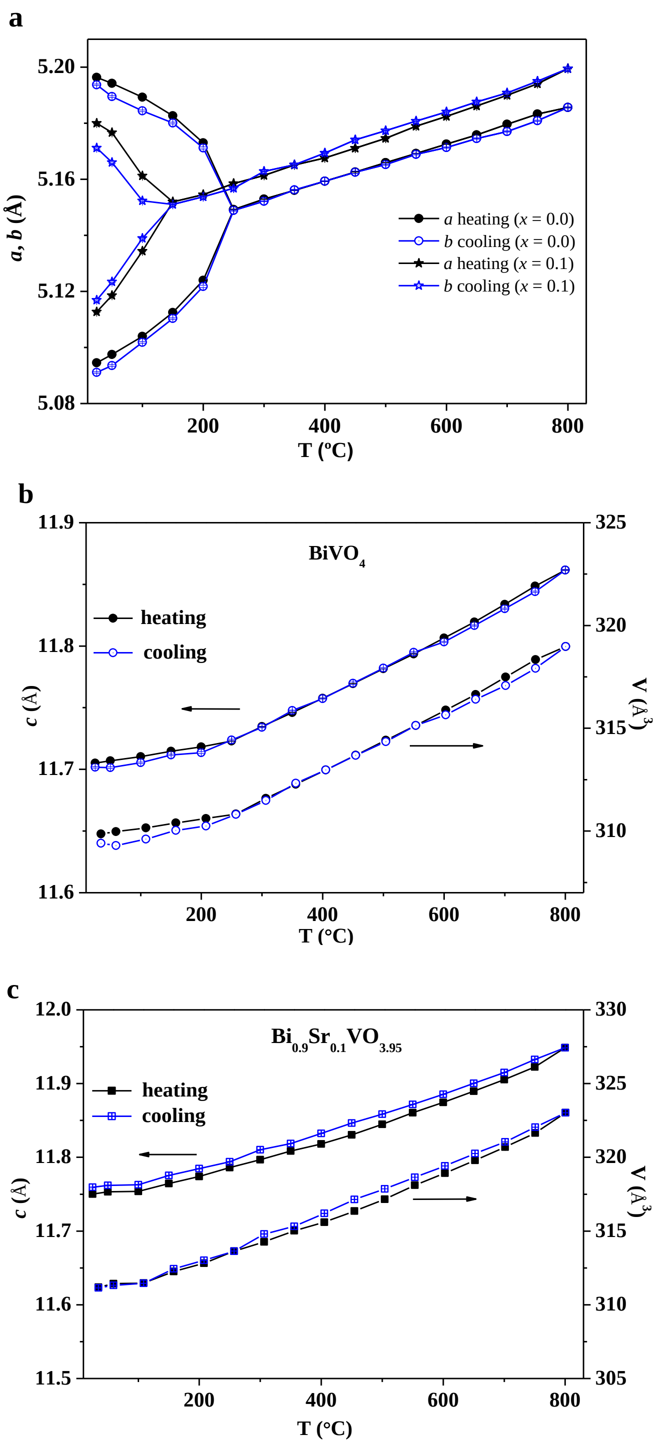

Supplementary Figure 7. Evolution of the lattice parameters with temperature. (a) Temperature dependence of the *a* and *b* cell parameters for $BiVO_4$ ($x = 0$) and $Bi_{0.9}Sr_{0.1}VO_{3.95}$ ($x = 0.1$) on heating and cooling. Below the phase transition temperature, the monoclinic phase shows anisotropic thermal expansion: *a* and *b* axes display negative and positive thermal expansions respectively and converged to the same value in the tetragonal phase. (b) and (c) show the thermal evolution of *c* and *V* cell parameters for $BiVO_4$ and $Bi_{0.9}Sr_{0.1}VO_{3.95}$ respectively.

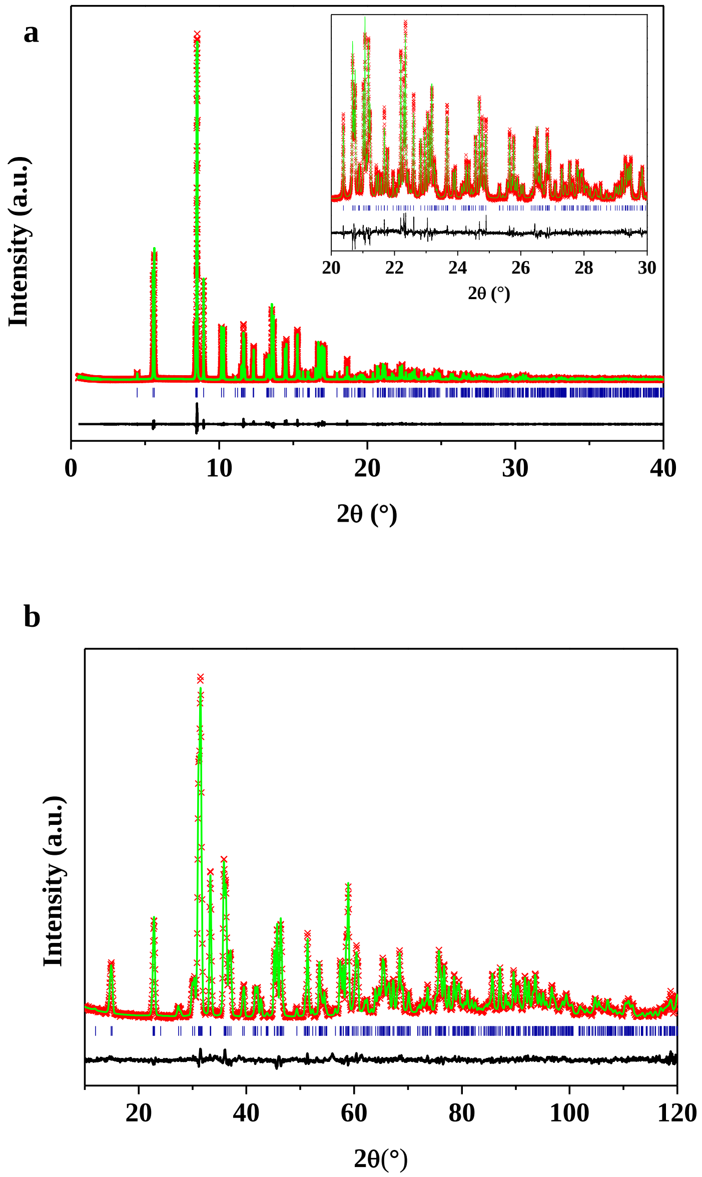

Supplementary Figure 8. Structure refinement using SPD and NPD data. Rietveld plots of (a) SPD and (b) NPD refinements for $Bi_{0.9}Sr_{0.1}VO_{3.95}$. The inset in (a) enlarges the fit of the SPD data within 20-30° 2θ region. The reliability factors are: R_p ~ 8.59 %; R_{wp} ~ 11.21%; GOF ~ 2.02 on SPD data; R_{wp} ~ 2.58%, R_p~ 2.07%, *GOF* ~ 1.54 on NPD data.

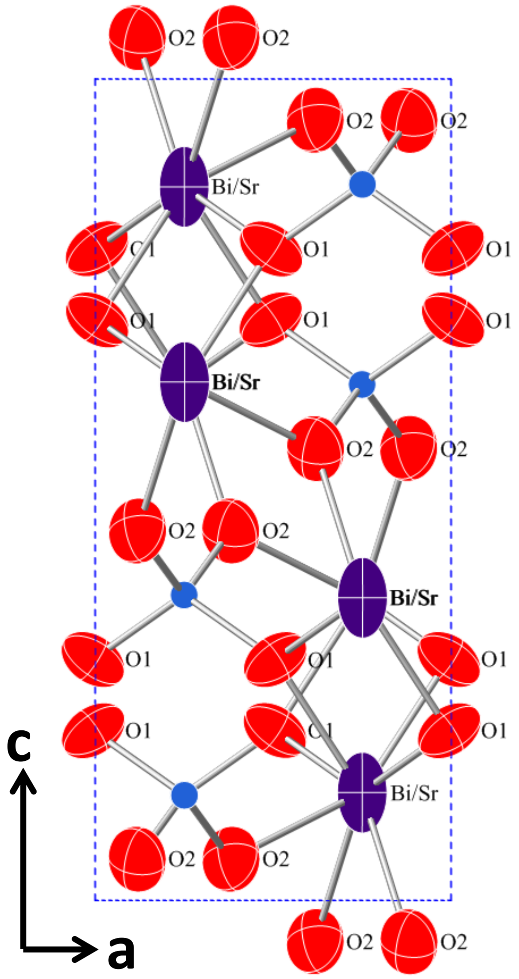

Supplementary Figure 9. Positional disorder induced by the oxygen vacancies. Ellipsoid view of the $Bi_{0.9}Sr_{0.1}VO_{3.95}$ refined structure from Rietveld Refinement of the NPD data along the [010] direction. The thermal ellipsoids have been drawn with a 99% presence probability. The ADPs for Sr/Bi and O atoms only were refined while the V atomic positional parameters were fixed at those from the refinement of SPD data.

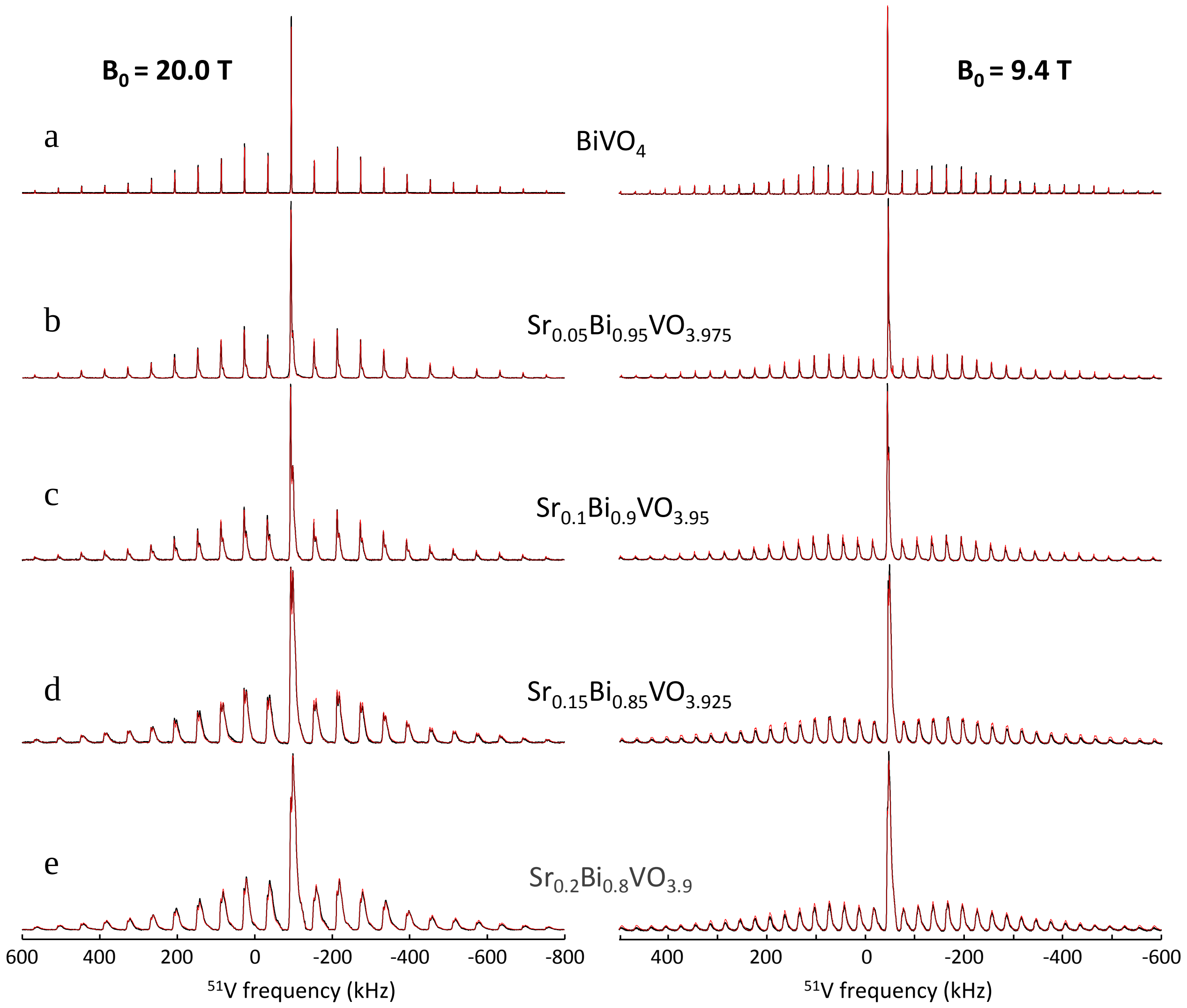

Supplementary Figure 10. ^{51}V MAS NMR Spectra. ^{51}V MAS NMR spectra of $Bi_{1-x}Sr_xVO_{4-0.5x}$ compounds (black lines) and their best fits (red lines) for compositions x = (a) 0, (b) 0.05, (c) 0.1, (d) 0.15 and (e) 0.2. The spectra recorded at 20.0 T (spinning frequency of 60 kHz) are shown on the left and those obtained at 9.4 T (spinning frequency of 30 kHz) are shown on the right.

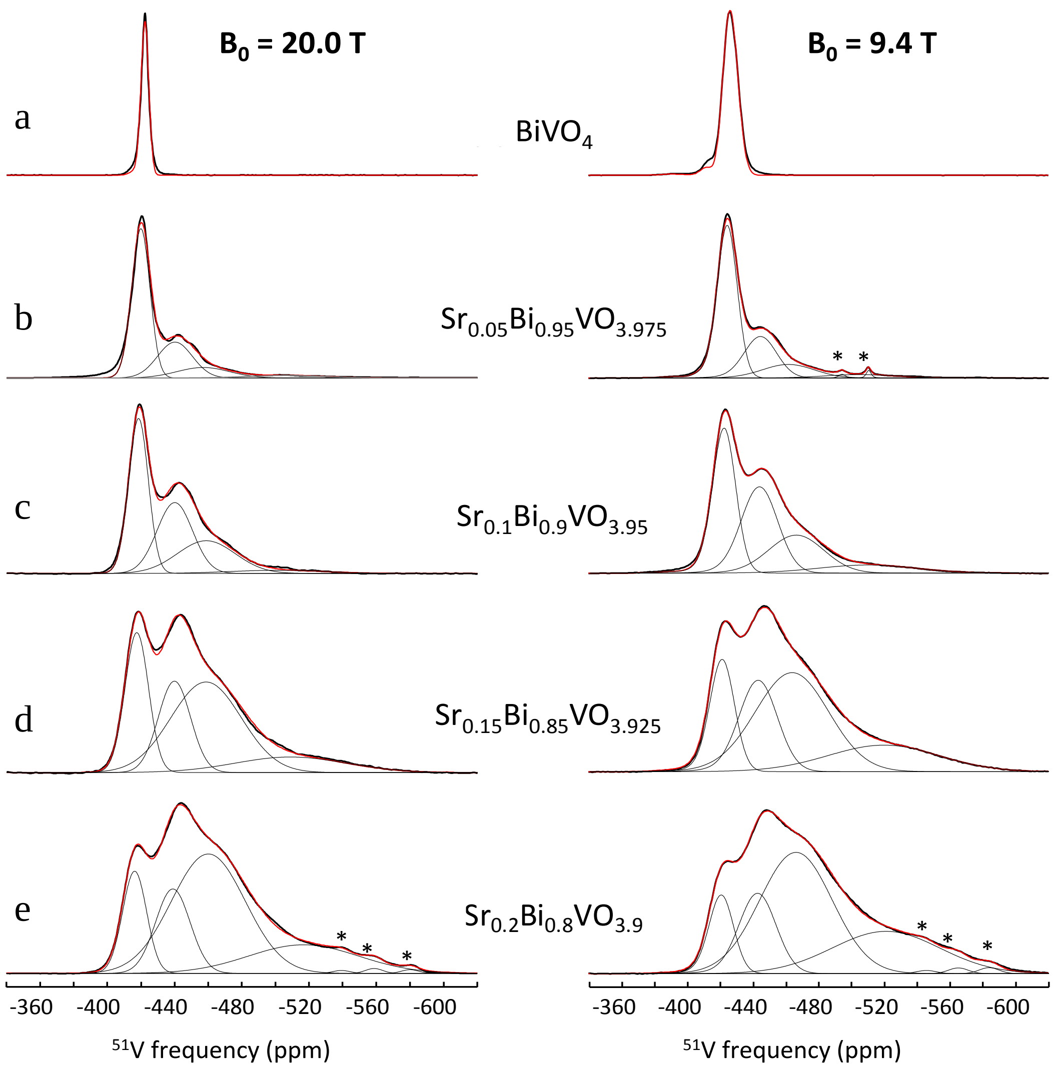

Supplementary Figure 11. Enlargement of ^{51}V MAS NMR Spectra. Expansion of the central transition region of the ^{51}V MAS NMR spectra of $Bi_{1-x}Sr_xVO_{4-0.5x}$ compounds. Experimental spectra and their best fits are shown in black and red, respectively. Individual contributions are indicated by the thin black lines and the asterisks indicate impurities. Spectra recorded at 20.0 T (spinning frequency of 60 kHz) are shown on the left and those obtained at 9.4 T (spinning frequency of 30 kHz) are shown on the right.

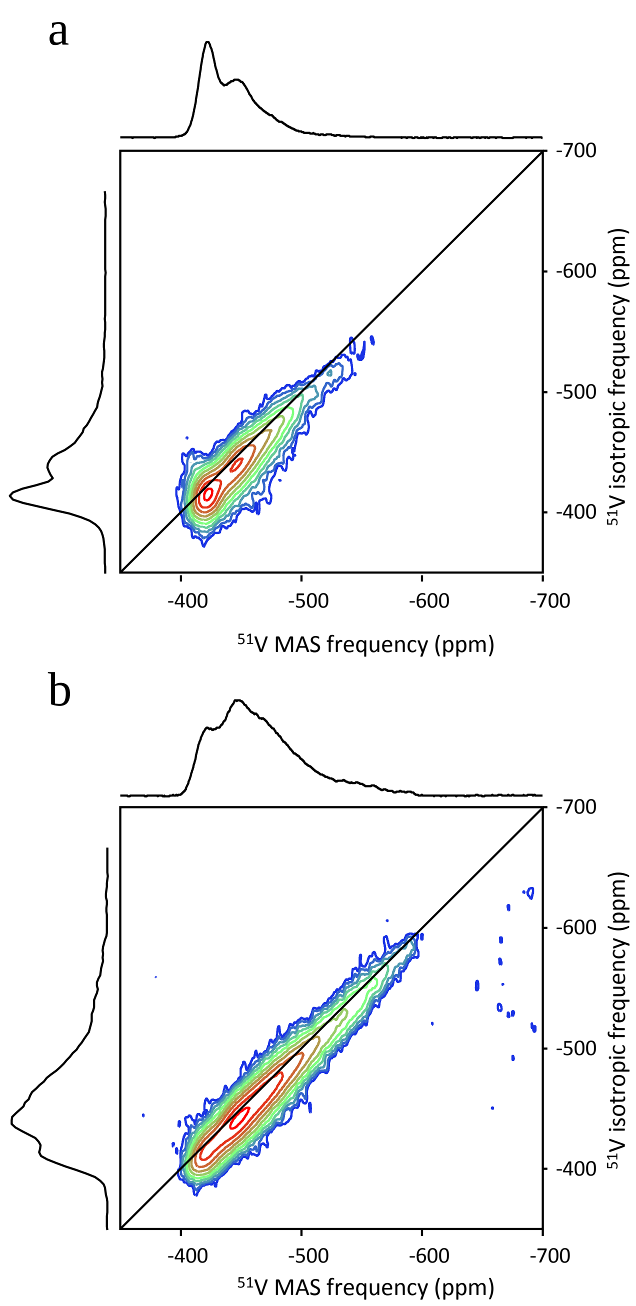

Supplementary Figure 12. 2D ^{51}V MQMAS spectra. 2D ^{51}V MQMAS spectra of (a) $Sr_{0.1}Bi_{0.9}VO_{3.95}$ and (b) $Sr_{0.2}Bi_{0.8}VO_{3.9}$ recorded at 9.4 T with a spinning frequency of 30 kHz. The diagonal (black line) of the 2D plot corresponds to isotropic chemical shift distribution axis.

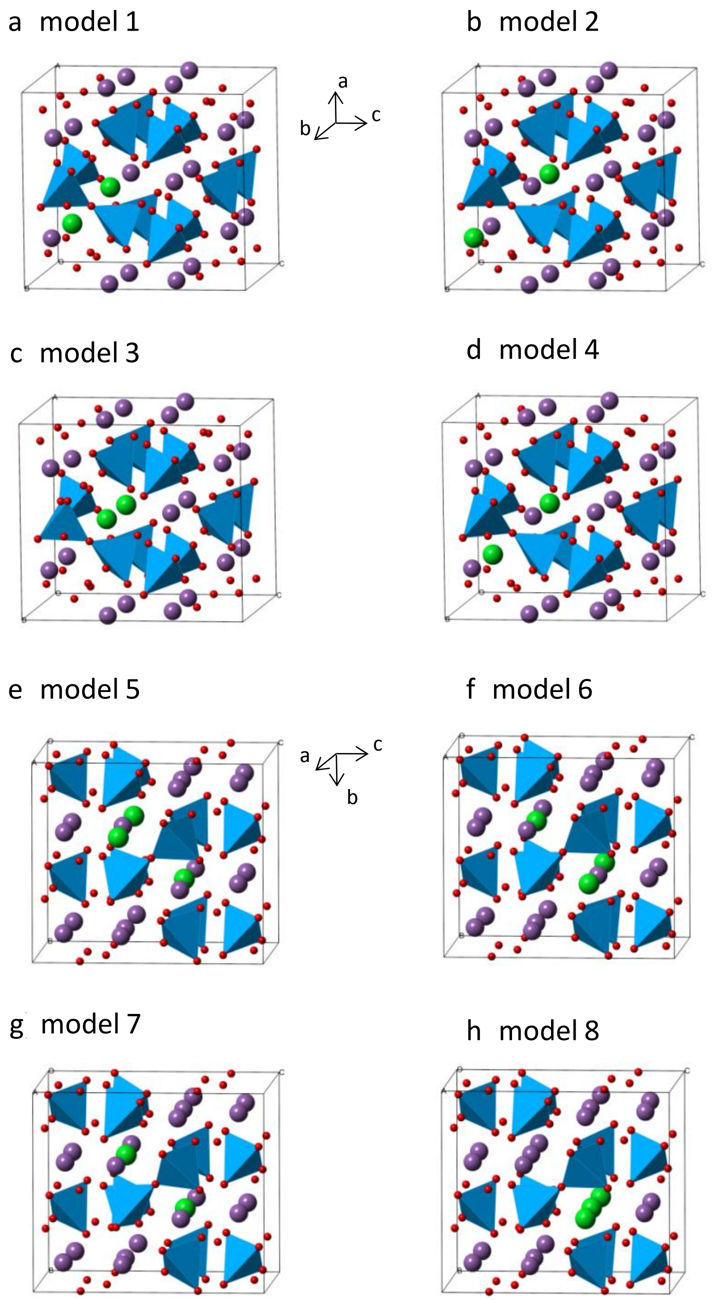

Supplementary Figure 13. 2×2×1 supercell models. 2×2×1 (geometry optimized) supercell models of $Sr_{0.125}Bi_{0.875}O_{3.9375}$. The models accommodate one oxygen vacancy and two Sr cations through the formation of a V_2O_7 unit, which were used for GIPAW computations of the ^{51}V NMR parameters. The violet, green and red spheres correspond to Bi, Sr and O atoms.

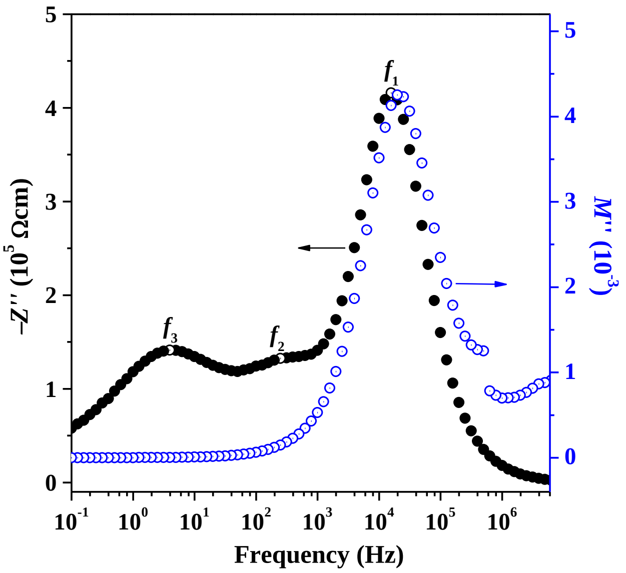

Supplementary Figure 14. *Z ''*~*f* and *M ''* ~*f* plots. Imaginary impedance Z′′ and modulus *M ''* as function of frequency at 300 °C for the $BiVO_4$ pellet. f_1 denotes the frequency associated with the *Z ''* and *M ''* peaks for the bulk response. The relaxation time τ for the bulk response arc, calculated according to equation $\tau = (2\pi f_1)^{-1}$, is 1.0042 $\times 10^{-5}$ s. f_2 and f_3 denote the frequencies corresponding to the maximum *Z ''* in the grain boundary and electrode response semicircular arcs, respectively.

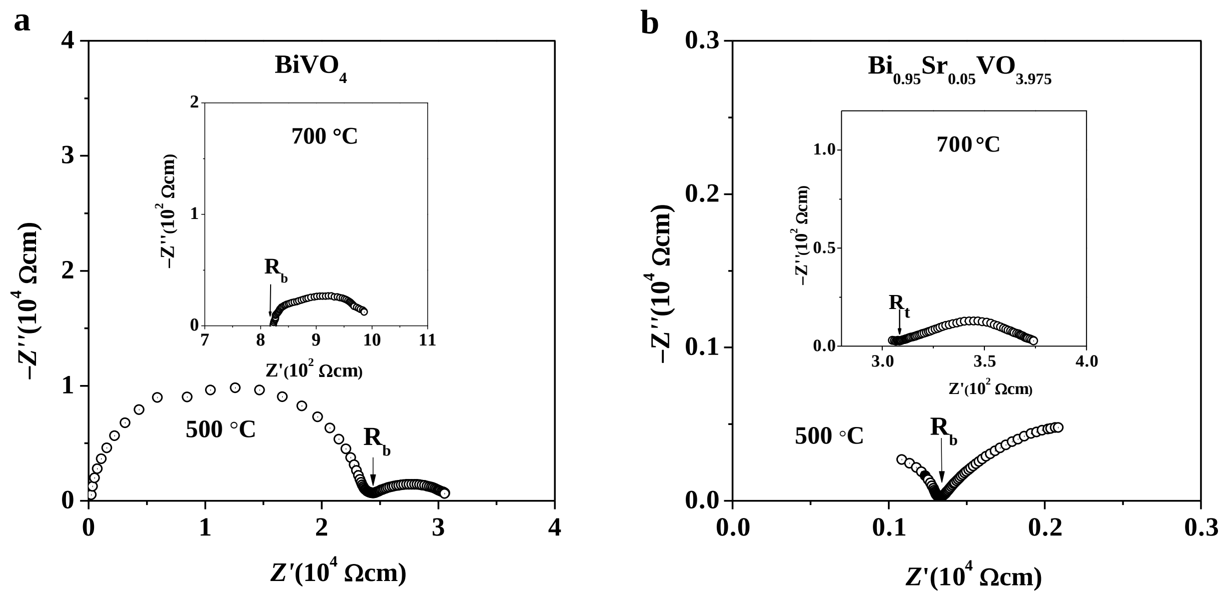

Supplementary Figure 15. Impedance behavior at elevated temperature. The Complex impedance plots of (a) $BiVO_4$ and (b) $Bi_{0.9}Sr_{0.1}VO_{3.95}$ materials recorded at 500 C and 700 °C (insets) in air. For $BiVO_4$, the grain boundary and electrode response arcs heavily overlap with each other all through the measured temperature range. While for $Bi_{0.9}Sr_{0.1}VO_{3.95}$, both bulk and grain boundary response arcs gradually disappear with the increase of temperature and the small grain boundary response arc became hardly discernable at high temperature. The intercept of the electrode response arc at high frequency for the total resistivity can be estimated as the bulk resistivity.

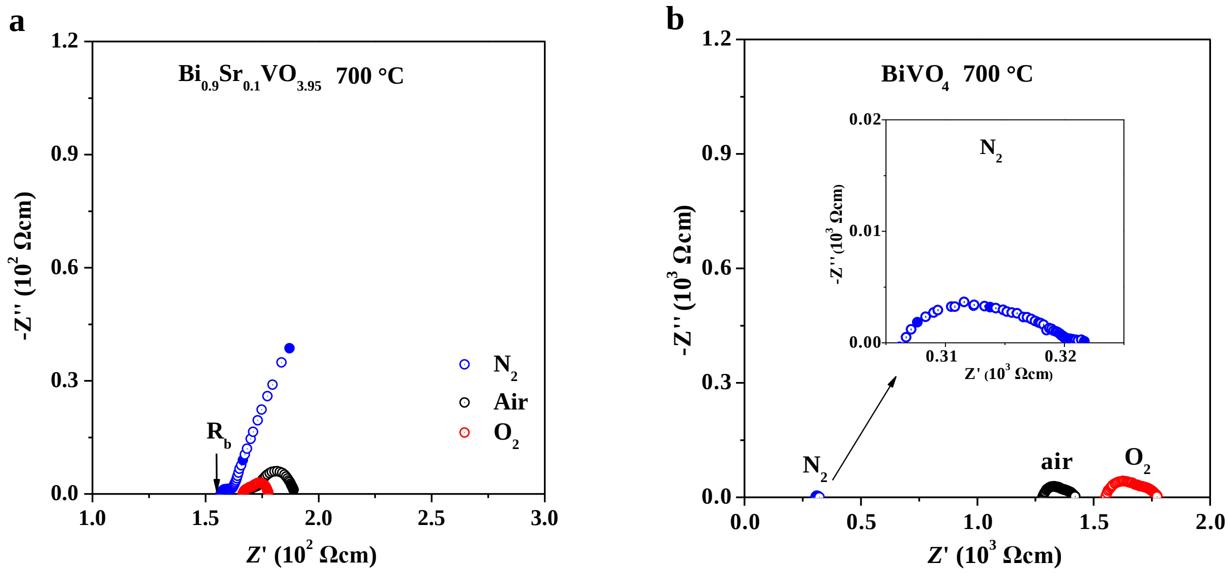

Supplementary Figure 16. Impedance behavior under different atmospheres. Complex impedance plots of (a) $Bi_{0.9}Sr_{0.1}VO_{3.95}$ and (b) $BiVO_4$ at 700 °C under N_2, air and O_2. The inset in (b) enlarges the plot under N_2 flow. Note that the resistance values for the substituted material are one order of magnitude smaller than those for the pristine one and the shape of electrode response arc for the pristine material is less affected by the pO_2.

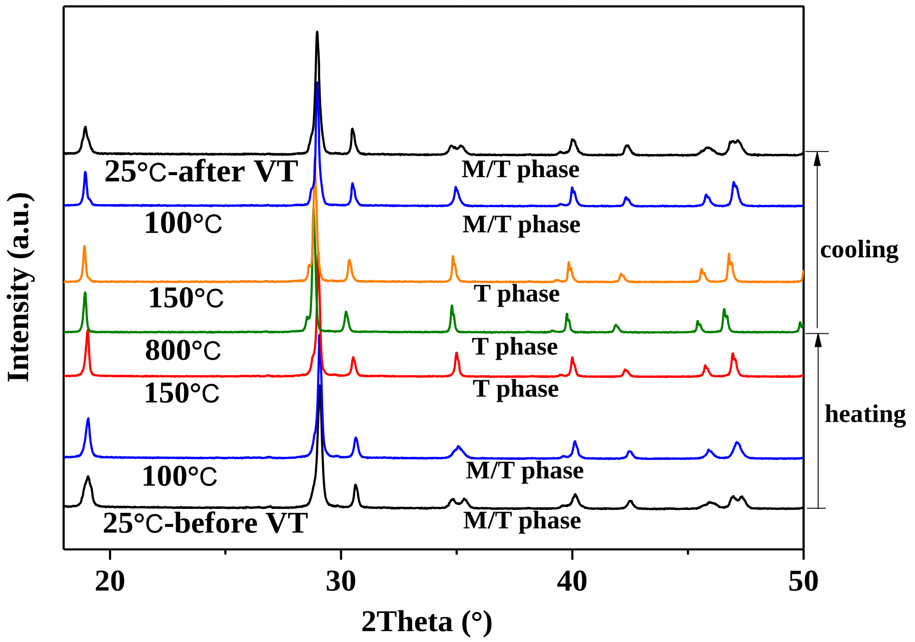

Supplementary Figure 17. VTXRD data of $Bi_{0.9}Sr_{0.1}VO_{3.95}$. Selected VTXRD patterns of the $Bi_{0.9}Sr_{0.1}VO_{3.95}$ sample synthesized by the CSS method. The data were recorded between 25 and 800°C, during heating and cooling. M and T denote monoclinic and tetragonal Scheelite phases, respectively.

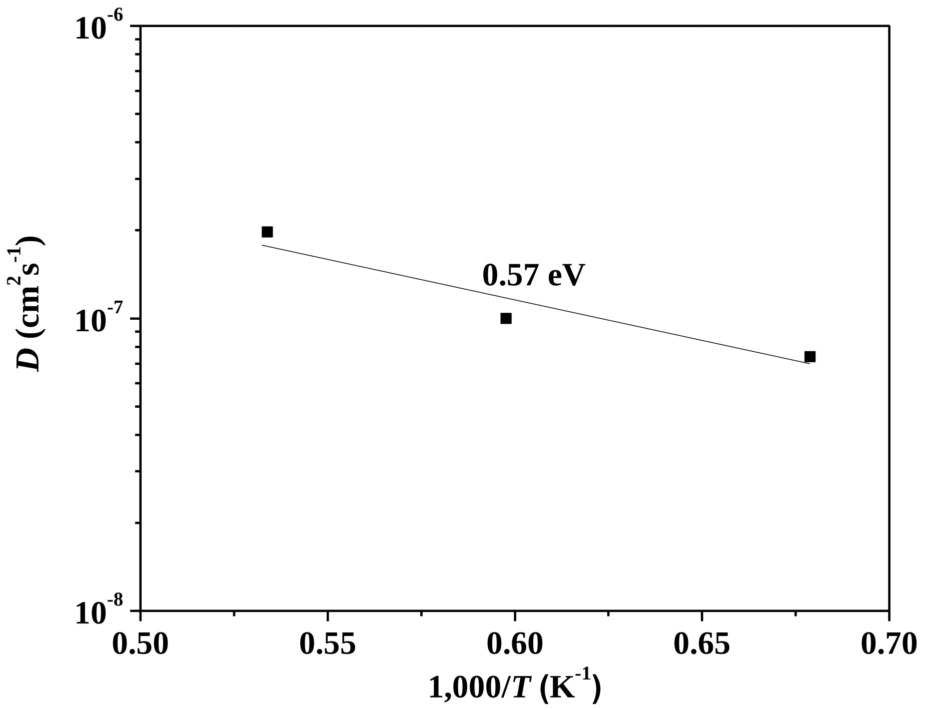

Supplementary Figure 18. Oxygen diffusion coefficient. Arrhenius plot of the oxygen diffusion coefficient calculated using the MSD values of oxygen atoms within 1200–1600 °C temperature range. The activation energy is labeled.

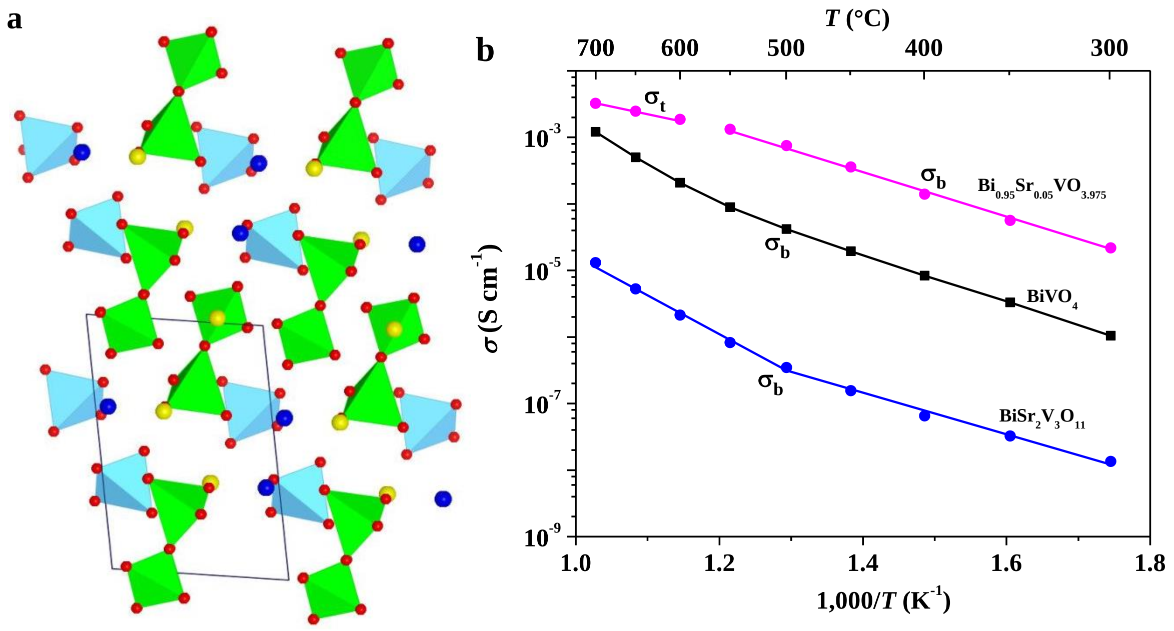

Supplementary Figure 19. Structure and Conductivity of $BiSr_2V_3O_{11}$. (a) View of crystal structure along the [001] direction and (b) conductivity of $BiSr_2V_3O_{11}$ containing isolated VO_4 units (tetrahedra in cyan) and V_2O_7 dimers (tetrahedra in green). The yellow and blue spheres in (a) denote Sr and Bi atoms, respectively.

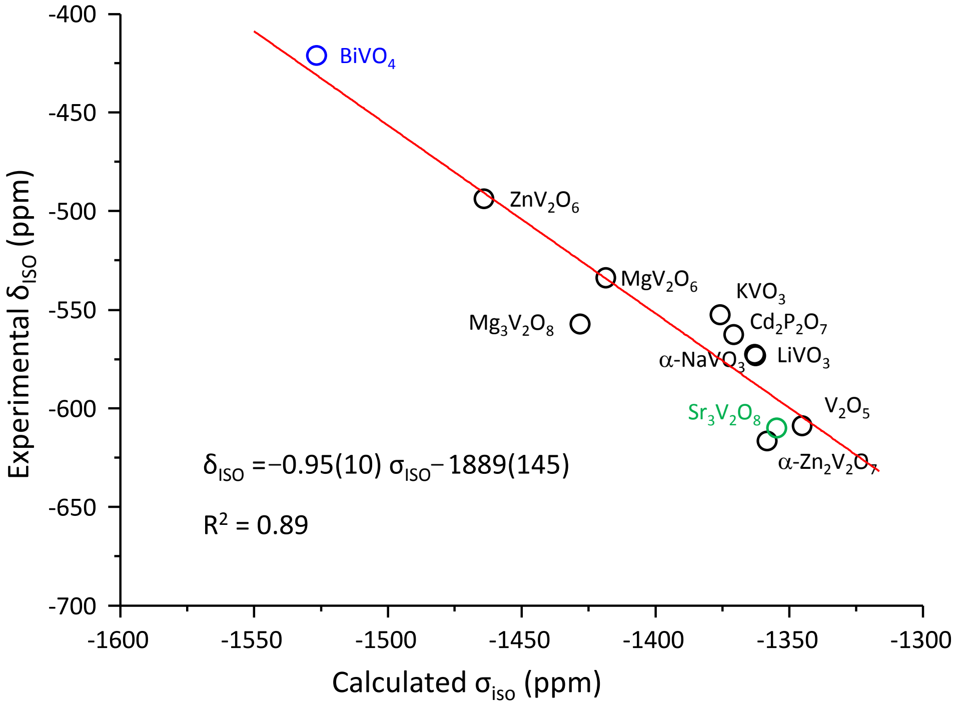

Supplementary Figure 20. Relationship between ^{51}V isotropic magnetic shieldings and isotropic chemical shifts. GIPAW-calculated ^{51}V isotropic magnetic shieldings (σ_{ISO}) *versus* experimentally measured ^{51}V isotropic chemical shifts (δ_{ISO}) for a series of crystalline vanadates (blue and green circle correspond to $BiVO_4$ and $Sr_3V_2O_8$ respectively). DFT optimization of the atomic positions, keeping symmetry constraints with fixed unit cell parameters, was performed prior GIPAW computations. The red line corresponds to the calculated linear regression (δ_{ISO} = –0.95 σ_{ISO} – 1889, R^2 = 0.89).

Supplementary Tables

Supplementary Table 1. EDS elementary analysis. EDS analysis of Bi, Sr and V in $Bi_{0.9}Sr_{0.1}VO_{3.95}$ synthesized by the ADL method. EDS data were recorded at 10 different regions. Nominal composition: 45 at.% Bi, 5 at.% Sr, 50 at.% V.

Spectrum Nº	Bi (at.%)	Sr (at.%)	V(at.%)
1	46.6	4.8	48.6
2	46.9	4.9	48.3
3	46.6	4.9	48.5
4	46.6	4.7	48.7
5	47.6	4.5	47.9
6	46.0	5.1	48.9
7	46.7	5.0	48.3
8	46.6	5.3	48.1
9	46.5	5.0	48.6
10	46.4	4.6	48.9
**Average Value**	**46.7(4)**	**4.9(2)**	**48.5(3)**

Supplementary Table 2. Refined Structural parameters. Atomic coordinates and isotropic atomic displacement parameters of $Bi_{0.9}Sr_{0.1}VO_{3.95}$ from Rietveld refinement of the SPD data.[a]

**Atom**	**Site**	***x***	***y***	***z***	**U_{iso} (Å^2)**	**Occupancy**
Bi	4*e*	0	0.25	0.63128(3)	0.01824(9)	0.893(2)
Sr	4*e*	0	0.25	0.63128(3)	0.01824(9)	0.107 (2)
V	4*e*	0	0.25	0.1283(1)	0.0142(4)	1
O1	8*f*	0.1466(6)	0.5098(5)	0.2069(2)	0.0166(9)	1
O2	8*f*	0.2584(5)	0.3856(6)	0.4485(3)	0.0177(9)	1

[a] Space group *I*2/*b*, *a* = 5.19161(1) Å, *b* = 5.11002(1) Å, *c* = 11.76879(4) Å, γ = 90.3835(2)°, *V* = 312.210(2) Å^3.

Supplementary Table 3. Refined Structural parameters. Refined structural parameters of $Bi_{0.9}Sr_{0.1}VO_{3.95}$ from Rietveld Refinement of NPD data. [a]

**Atom**	**Site**	***x***	***y***	***Z***	**U_{iso} (Å^2)**	**Occupancy**
Bi	4*e*	0	0.25	0.6313(1)*	0.0188(4)	0.893(2)*
Sr	4*e*	0	0.25	0.6313(1)*	0.0188(4)	0.107(2)*
V	4*e*	0	0.25	0.1283(1)*	0.0142(4)*	1
O1	8*f*	0.1422(2)	0.5059(2)	0.2067(1)	0.0146(4)	0.973(1)
O2	8*f*	0.2577(2)	0.3813(3)	0.4498(1)	0.0171(4)	0.986(1)

**Atom**	**U_{11}(Å^2)**	**U_{22}(Å^2)**	**U_{33}(Å^2)**	**U_{12}(Å^2)**	**U_{13}(Å^2)**	**U_{23}(Å^2)**
Bi	0.0153(7)	0.0113(7)	0.0297(9)	0.0001(6)	0	0
Sr	0.0153(7)	0.0113(7)	0.0297(9)	0.0001(6)	0	0
V[b]	-	-	-	-	-	-
O1	0.0144(8)	0.0151(8)	0.0141(9)	−0.0025(5)	0.0014(6)	−0.0062(8)
O2	0.0151(8)	0.0162(8)	0.0197(9)	0.013(6)	0.0043(7)	−0.0005(6)

[a] space group *I*2/*b*, a = 5.1915(1) Å, b = 5.1116(1) Å, c = 11.7727(3) Å, γ = 90.380(2)°, V = 312.41(2) Å^3. *Values fixed at those from SPD data refinement (Table S5). [b] The ADPs for V site were not refined owing to the low sensitivity of neutrons to vanadium.

Supplementary Table 4. ^{51}V **NMR parameters.** ^{51}V isotropic chemical shift (δ_{ISO}), quadrupolar coupling constant ($|C_Q|$), biaxiality of the EFG tensor (η_Q), full-width at half maximum (fwhm) and relative intensities (I) of the four individual contributions in ^{51}V MAS NMR spectra of $Bi_{1-x}Sr_xVO_{4-0.5x}$ samples recorded at 20.0 T and 9.4 T.

Compound	Unit	δ_{ISO} (ppm)	$\|C_Q\|$ MHz	η_Q	fwhm (ppm)	I (% ± 5)
$Sr_{0.05}Bi_{0.95}VO_{3.975}$	VO_4 (0Sr, 8Bi)	−419(1)	4.83(2))	0.4(1)	12.5(5)	59
	VO_4 (1Sr, 7Bi)	−440(4)	4.54(8)	0.5(1)	23(1)	26
	VO_4 (2Sr, 6Bi)	−456(5)	4.4(1)	0.6(2)	37(3)	12
	V_2O_7	−500(10)	4.6(2)	0.6(2)	50(10)	3
$Sr_{0.1}Bi_{0.9}VO_{3.95}$	VO_4 (0Sr, 8Bi)	−417(1)	4.75(5)	0.4(1)	14(1)	37
	VO_4 (1Sr, 7Bi)	−439(3)	4.5(1)	0.5(1)	25(1)	33
	VO_4 (2Sr, 6Bi)	−459(5)	4.4(2)	0.6(2)	40(2)	24
	V_2O_7	−500(10)	4.6(2)	0.6(2)	66(8)	6
$Sr_{0.15}Bi_{0.95}VO_{3.925}$	VO_4 (0Sr, 8Bi)	−416(1)	4.70(5)	0.4(1)	16(1)	20
	VO_4 (1Sr, 7Bi)	−438(2)	4.4(1)	0.4(1)	24(2)	21
	VO_4 (2Sr, 6Bi)	−458(5)	4.4(2)	0.6(2)	50(4)	43
	V_2O_7	−510(10)	4.6(4)	0.7(2)	70(5)	16
$Sr_{0.2}Bi_{0.8}VO_{3.9}$	VO_4 (0Sr, 8Bi)	−415(1)	4.7(1)	0.4(1)	15(1)	13
	VO_4 (1Sr, 7Bi)	−438(2)	4.4(1)	0.4(1)	26(2)	17
	VO_4 (2Sr, 6Bi)	−462(5)	4.4(2)	0.4(2)	51(4)	48
	V_2O_7	−520(5)	4.7(3)	0.8(2)	70(10)	21

Supplementary Table 5. Interatomic potential parameters for Sr-substituted $BiVO_4$ Scheelite.

(a) Short-range

**Interaction**	**A (eV)**	**ρ (Å)**	**C (eV Å^6)**
Bi^{3+}-O^{2-} [3]	49529.35	0.2223	0
Bi^{3+}-Bi^{3+}	85839.392	0.3284	0
Sr^{2+}-O^{2-} [4]	1400	0.35	0
V^{5+}-O^{2-} [5]	668.87	0.4095	0
O^{2-}-O^{2-}	20117.481	0.2192	32

(b) Shell model

**Interaction**	**Y (e)**	***k* (eV Å^{-2})**
Bi^{3+}	–5.51	359.55
O^{2-}	−2.04	6.3
Sr^{2+}	1.33	21.53

Supplementary Table 6. Calculated and experimental structural parameters for $BiVO_4$.

Parameters	Calculated	Experimental	Δ(Calc. −Exp.)
a (Å)	5.142	5.197	–0.055
b (Å)	5.142	5.096	0.046
c (Å)	11.702	11.702	0
γ (°)	90.0	90.4	–0.4
V-O2(×2) (Å)	1.677	1.744	–0.067
V-O1(×2) (Å)	1.677	1.747	–0.07
Mean V-O (Å)	1.677	1.746	–0.069
Bi-O1(×2) (Å)	2.545	2.346	0.199
Bi-O1(×2) (Å)	2.449	2.375	0.074
Bi-O2(×2) (Å)	2.449	2.528	–0.079
Bi-O2(×2) (Å)	2.545	2.606	–0.061
Mean Bi-O (Å)	2.497	2.464	0.033

Supplementary Table 7. Pseudopotentials parameters. Description of the pseudopotentials used for the planewave-based DFT calculations with the CASTEP code. r_{loc} is the pseudisation radius for the local component of the pseudopotential, r_{nonloc} is the pseudisation radius for the non-local components of the pseudopotential, and r_{aug} is the pseudisation radius for the charge augmentation functions.

Atom	Core-states	r_{loc}	r_{nonloc}	r_{aug}	USPP projectors
**O**	1s	1.1	1.1	0.77	2×2s, 2×2p
**V**	1s, 2s, 2p	2.0	2.0	1.0	1×3s, 2×3p, 2×3d, 2×4s
**Sr**	1s, 2s, 2p, 3s, 3p, 3d	2.0	2.0	1.4	1×4s, 2×4p, 2×4d, 2×5s
**Bi**	1s, 2s, 2p, 3s, 3p, 3d, 4s, 4p, 4d, 4f, 5s, 5p	2.3	2.3	1.6	2×5d, 2×6s, 2×6p
Pseudopotentials used for reference crystalline compounds					
**Li**		1.0	1.0	0.7	1×1s, 2×2s
**Na**	1s	1.3	1.3	0.9	1×2s, 2×2p, 2×3s
**Mg**	1s	1.1	1.1	0.77	2×2s, 2×2p
**K**	1s, 2s, 2p	1.5	1.5	1.0	1×3s, 2×3p, 2×4s
**Zn**	1s, 2s, 2s, 3s, 3p	2.0	2.0	1.0	2×3d, 2×4s, 2×2p
**Cd**	1s, 2s, 2p, 3s, 3p, 3d, 4s, 4p	2.2	2.2	1.53	2×4d, 2×5s

Supplementary Table 8. ^{51}V isotropic magnetic shieldings and isotropic chemical shifts. Experimental ^{51}V isotropic chemical shift (δ_{ISO}) and GIPAW calculated isotropic shielding values (σ_{ISO}) for some reference compounds. DFT atomic position optimization (keeping symmetry constraint and constraining cell parameters to experimental values) was performed before GIPAW computations. All computations were performed using the PBE generalized gradient approximation, ultrasoft pseudopotentials (USPP) described in supplementary Table 7, a kinetic energy cut-off of 600 eV and a Monkhorst-Pack grid spacing of 0.04 $Å^{-1}$.

**Compounds**	**Unit**	**δ_{ISO}^{exp} (ppm)**	**Ref.**	**σ_{ISO}^{calc} (ppm)**
$BiVO_4$	VO_4 (isolated)	−421.1	(6)	−1526.6
$Sr_3V_2O_8$	VO_4 (isolated)	−610	(7)	−1354.7
$Mg_3V_2O_8$	VO_4 (isolated)	−557.3	(6)	−14428.1
α-$Zn_2V_2O_7$	VO_4 (dimer)	−616.6	(8)	−1358.2
$Cd_2V_2O_7$	VO_4 (dimer)	−562.7	(8)	−1370.7
MgV_2O_6	VO_4 (chain)	−533.9	(9)	−1418.5
ZnV_2O_6	VO_4 (chain)	−493.8	(9)	−1464.0
$LiVO_3$	VO_4 (chain)	−573.4	(10)	−1362.9
α-$NaVO_3$	VO_4 (chain)	−572.7	(10)	−1362.9
KVO_3	VO_4 (chain)	−552.7	(10)	−1375.8
V_2O_5	VO_5	−609	(11)	−1345.1

Supplementary Notes

Supplementary Note 1. $Bi_{1-x}Sr_xVO_{4-0.5x}$ solid solution from CSS route

Supplementary Fig. 1 shows ambient temperature XRD data of $Bi_{1-x}Sr_xVO_{4-0.5x}$ samples synthesized from the CSS method. The analysis of their XRD patterns indicates that Sr-substituted compositions (x = 0.05–0.1) form a mixture of monoclinic (m-$Bi_{1-x}Sr_xVO_{4-0.5x}$) and tetragonal (t-$Bi_{1-x}Sr_xVO_{4-0.5x}$) Scheelite phases. This is evidenced by a characteristic set of extra reflections (Supplementary Fig. 1–2), along with the presence of $BiSr_2V_3O_{11}$ as a secondary phase for compositions $x > 0.1$. The refined cell parameters of monoclinic and tetragonal Scheelite phases from a two-phase Rietveld refinement (Supplementary Fig. 2) of the $Bi_{0.9}Sr_{0.1}VO_{3.95}$ XRD data are: a = 5.1880(1) Å, b = 5.1083(1) Å, c = 11.7507(3) Å, β = 90.359(1)°, V = 311.41(1) Å^3 for the monoclinic phase with I2/b space group (~ 80 wt%) and a = 5.1469(1) Å, c = 11.7545(4) Å, V = 311.39(2) Å^3 for the tetragonal phase with $I4_1/a$ symmetry (~ 20 wt%). The close cell volumes of the monoclinic and tetragonal phases indicate close compositions for both phases. This is confirmed by EDS point measurements and elemental mappings showing homogenous composition of the Scheelite phases in the $Bi_{0.9}Sr_{0.1}VO_{3.95}$ sample (Supplementary Fig. 3). Therefore the monoclinic and tetragonal phases are different polymorphs with the same composition. Various thermal treatments were attempted in order to obtain single-polymorph $Bi_{1-x}Sr_xVO_{4-0.5x}$, including annealing in oxygen atmosphere or within the 100–130 °C temperature range, i.e. below the phase transition temperature, as well as quenching from high temperature (500–700 °C). However, presence of the tetragonal polymorph cannot be avoided using these approaches.

Supplementary Note 2. Impedance data analysis

The deconvolution of the impedance plot for $BiVO_4$ at 300 °C (Figure 5a) was performed through the equivalent circuit fitting with bulk, grain boundary and electrode components in a serial combination. Simply three parallel R_i, C_i and $(CPE)_i$ elements (i may be b for bulk, gb for grain boundary and e for electrode, CPE is a constant phase element) are used to model each component for the deconvolution. The fit gave R_b ~ 9.54×10^5 Ω cm, C_b ~ 10.02 pF cm^{-1}, R_{gb} ~ 2.03×10^5 Ωcm, C_{gb} ~ 2.16 nF cm^{-1}, and R_e ~ 5.49×10^5 Ω cm, C_e ~ 2.23×10^{-8} F cm^{-1}. The impedance data analysis for the $Bi_{0.95}Sr_{0.05}VO_{3.975}$ composition at 300 °C follows the same procedure as the one described in the main text for pristine $BiVO_4$. The capacitance values for the large semicircular arc (Fig. 5b) of $Bi_{0.95}Sr_{0.05}VO_{3.975}$, calculated using equations of $\omega RC = 1$ and $M''_{max} = \varepsilon_0/2C$, are 13 pF cm^{-1} and 14 pF cm^{-1}, confirming that the large semicircular arc can be ascribed to single bulk response. The equivalent circuit fitting was performed using an equivalent circuit consisting of two circuits with three parallel R_i, C_i, $(CPE)_i$ elements corresponding to the bulk and grain boundary responses and one Warburg element for the electrode response in a serial combination. The fit gave R_b ~ 4.53×10^4 Ω cm, C_b ~ 14.8 pF cm^{-1}, R_{gb} ~ 1.48×10^4 Ω cm, C_{gb} ~ 3.85×10^{-10} F cm^{-1} and an exponent parameter ~ 0.54 for the Warburg element. It should be noted here that the electrode responses in both $BiVO_4$ and Sr-doped $BiVO_4$ are much more complex than the simple circuits used here as the ion diffusion, electronic conduction even the double electrical layers and charge transfer reaction along the sample-electrode interface are involved in the mixed electronic and oxide ionic conducting $BiVO_4$ and Sr-doped $BiVO_4$. However, these equivalent circuit fittings using the simplified circuits for the electrode responses do not affect significantly the reported bulk and total conductivities here for $Bi_{1-x}Sr_xVO_{4-0.5x}$.

Supplementary Video.

The video file illustrating the oxygen vacancy migration in $BiVO_4$ via the cooperative mechanism.[2]

Supplementary References

1. Stephens, P. W. Phenomenological model of anisotropic peak broadening in powder diffraction. *J. Appl. Crystallogr.* **32**, 281–289 (1999).

2. Allix, M. et al. Highly Transparent $BaAl_4O_7$ Polycrystalline Ceramic Obtained by Full Crystallization from Glass. *Adv. Mater.* **24**, 5570–5575 (2012).

3. Abrahams, I. et al. Combined Total Scattering and Simulation Approach to Analyzing Defect Structure in Bi_3YO_6. *Chem. Mater.* **22**, 4435–4445 (2010).

4. Lewis, G. V. & A Catlow C. R. Potential models for ionic oxides. *J. Phys. C: Solid State Phys.* **18**, 1149–1161 (1985).

5. Jr, B. F. D. S., Araujo R. M., Valerio M. E. G. & Rezende M. V. D. S. Optical spectroscopy study of YVO_4:Eu^{3+} nanopowders prepared by the proteic sol–gel route. *Solid State Sci.* **42**, 45–51 (2015).

6. Skibsted, J., Jacobsen C. J. H. & Jakobsen H. J. ^{51}V Chemical Shielding and Quadrupole Coupling in Ortho- and Metavanadates from ^{51}V MAS NMR Spectroscopy. *Inorg. Chem.* **37**, 3083–3092 (1998).

7. Lapina, O. B., Mastikhin V. M., Shubin A. A., Krasilnikov V. N. & Zamaraev K. I. ^{51}V Solid state NMR studies of vanadia based catalysts. *Prog. Nucl. Magn. Reson. Spectrosc.* **24**, 457–525 (1992).

8. Nielsen, U. G., Jakobsen H. J. & Skibsted J. ^{51}V MAS NMR investigation of ^{51}V quadrupole coupling and chemical shift anisotropy in divalent metal pyrovanadates. *J. Phys. Chem. B* **105**, 420–429 (2001).

9. Nielsen, U. G., Jakobsen H. J. & Skibsted J. Characterization of Divalent Metal Metavanadates by ^{51}V Magic-Angle Spinning NMR Spectroscopy of the Central and Satellite

Transitions. *Inorg. Chem.* **39**, 2135–2145 (2000).

10. Skibsted, J., Nielsen N. C., Bildsoe H. & Jakobsen H. J. Magnitudes and relative orientation of vanadium-51 quadrupole coupling and anisotropic shielding tensors in metavanadates and potassium vanadium oxide (KV_3O_8) from vanadium-51 MAS NMR spectra. Sodium-23 quadrupole coupling parameters for .alpha.- and .beta.-$NaVO_3$. *J. Am. Chem. Soc.* **115**, 7351–7362 (1993).

11. Skibsted, J., Nielsen N. C., Bildsøe H. & Jakobsen H. J. ^{51}V MAS NMR spectroscopy: determination of quadrupole and anisotropic shielding tensors, including the relative orientation of their principal-axis systems. *Chem. Phys. Lett.* **188**, 405–412 (1992).
