# Supplementary material for: Cooperative mechanisms of oxygen vacancy stabilization and migration in the isolated tetrahedral anion Scheelite structure
Source: Nat Commun. 2018 Oct 26;9:4484. doi: 10.1038/s41467-018-06911-w (PMC6203716; doi:10.1038/s41467-018-06911-w)
Supplement: Supplementary file 2 — Description of Additional Supplementary Files [file 41467_2018_6911_MOESM2_ESM.pdf]

.

Supplementary Video.

The video file illustrating the oxygen vacancy migration in $BiVO_4$ via the cooperative mechanism.
